# Systematic Review of Protein Biomarkers in Adult Patients With Chronic Rhinosinusitis

**DOI:** 10.1177/19458924231190568

**Published:** 2023-07-25

**Authors:** Shyam A. Gokani, Andreas Espehana, Ana C. Pratas, Louis Luke, Ekta Sharma, Jennifer Mattock, Jelena Gavrilovic, Allan Clark, Tom Wileman, Carl M. Philpott

**Affiliations:** 1 Norwich Medical School, 6106 University of East Anglia, Norwich, UK; 2 James Paget University Hospital, Gorleston, UK; 3 University College London Hospital, London, UK; 4 School of Biological Sciences, 6106 University of East Anglia, Norwich, UK; 5 7308 Quadram Institute Bioscience, Norwich, UK

**Keywords:** biomarkers, chronic rhinosinusitis, endotypes, nasal polyps, cytokines, CRSwNP, CRSsNP, ECRS, phenotypes, interleukin

## Abstract

**Background:**

Chronic rhinosinusitis (CRS) is a heterogeneous condition characterized by differing inflammatory endotypes. The identification of suitable biomarkers could enable personalized approaches to treatment selection.

**Objective:**

This study aimed to identify and summarize clinical studies of biomarkers in adults with CRS in order to inform future research into CRS endotypes.

**Methods:**

We conducted systematic searches of MEDLINE and Web of Science from inception to January 30, 2022 and included all clinical studies of adult CRS patients and healthy controls measuring biomarkers using enzyme-linked immunosorbent assays or Luminex immunoassays. Outcomes included the name and tissue type of identified biomarkers and expression patterns within CRS phenotypes. Study quality was assessed using the National Institutes of Health quality assessment tool for observational cohort and cross-sectional studies. A narrative synthesis was performed.

**Results:**

We identified 78 relevant studies involving up to 9394 patients, predominantly with CRS with nasal polyposis. Studies identified 80 biomarkers from nasal tissue, 25 from nasal secretions, 14 from nasal lavage fluid, 24 from serum, and one from urine. The majority of biomarkers found to distinguish CRS phenotypes were identified in nasal tissue, especially in nasal polyps. Serum biomarkers were more commonly found to differentiate CRS from controls. The most frequently measured biomarker was IL-5, followed by IL-13 and IL-4. Serum IgE, IL-17, pentraxin-3 and nasal phospho-janus kinase 2, IL-5, IL-6, IL-17A, granulocyte-colony stimulating factor, and interferon gamma were identified as correlated with disease severity.

**Conclusion:**

We have identified numerous potential biomarkers to differentiate a range of CRS phenotypes. Future studies should focus on the prognostic role of nasal tissue biomarkers or expand on the more limited studies of nasal secretions and nasal lavage fluid.

We registered this study in PROSPERO (CRD42022302787).

## Introduction

Chronic rhinosinusitis (CRS) is an inflammatory disorder of the nose and paranasal sinuses which persists for more than 12 weeks without resolution.^1^ CRS is estimated to affect 11% of the global population^1,2^ and an estimated one in three patients have poorly controlled symptoms in secondary care.^3,4^ Existing measures of disease activity, such as nasoendoscopy, disease specific quality of life scores, or computed tomography scans^1,5^ can be retrospective in nature. Changes in these measures are often apparent only after disease control has regressed significantly, so better modalities to predict treatment response are needed.

Biomarkers are characteristics which can be objectively measured as an indicator of biological processes or responses to a therapeutic intervention.^6^ Since CRS is a heterogeneous condition, the identification of suitable biomarkers for determining CRS endotypes and predicting treatment effectiveness will enable personalized approaches to treatment selection.^7,8^

CRS was traditionally classified into CRS with nasal polyps (CRSwNP) or CRS without nasal polyps (CRSsNP) however this approach does not capture the complexity of CRS phenotypes. The most recent European Position Paper on Sinusitis (EPOS2020) has set out type 2 and non-type 2 inflammation as two important endotypes of primary CRS.^1^ The type 2 endotype is characterized by phenotypes such as CRSwNP and eosinophilic CRS with nasal polyps (ECRSwNP), defined as a tissue eosinophil count of ≥10 per high powered field or blood eosinophils ≥250 cells per microliter.

Biologic agents for treating asthma such as omalizumab are increasingly being used for CRSwNP, which has established a need for improved subtyping of the disease to enhance treatment efficacy.^9^ Emerging evidence exists that CRS biomarkers are not mutually exclusive and can be used to determine CRS prognosis independently of phenotype.^10^ Therefore, the aim of this review was to identify and summarize clinical studies of biomarkers in adults with CRS in order to inform future research into CRS endotypes.

## Materials and Methods

### Search Protocol and Selection Criteria

The protocol for this review was designed according to the Preferred Reporting Items for Systematic Review and Meta-Analysis guidelines^11^ and was registered in PROSPERO (CRD42022302787). Systematic searches were initially performed of MEDLINE via Ovid SP and Web of Science from January 1, 2006 to October 1, 2018, and subsequently updated from inception to January 30, 2022 with no limits. The full search strategy is outlined in Supplemental file 1. Text-based synonyms and medical subject headings terms for the key search themes outlined in Table 1 were combined with Boolean operators. Additional studies were identified from the references of relevant studies and systematic reviews.

**Table 1.** Search Strategy.

PICO parameter	Description
Population	Adult patients (≥18 years of age) with CRS (clinical, endoscopic and/or radiological evidence of chronic inflammation of the nose and paranasal sinuses)
Intervention	Clinical measurement of biomarkers using ELISA or Luminex assays
Comparator	Healthy adult patients with no evidence of CRS or allergic rhinitis
Outcomes	Name of biomarker, tissue of biomarker origin, biomarker expression pattern within CRS phenotypes

All clinical studies of adult patients (≥18 years of age) with CRS and healthy controls which reported the measurement of biomarkers through enzyme-linked immunosorbent assay (ELISA) or Luminex were included. CRS was defined as clinical, endoscopic, and/or radiological evidence of chronic inflammation of the nose and paranasal sinuses. Studies of patients with all subtypes of CRS including primary and secondary CRS were eligible for inclusion. Healthy control patients were defined as adults (≥18 years of age) with no evidence of CRS or allergic rhinitis. Eligible study designs included randomized controlled trials and observational studies (including cohort and cross-sectional studies) so that all relevant data could be considered. Animal studies, in vitro studies, reviews, editorials, letters, and conference abstracts were excluded. Studies were excluded if they used ELISA or Luminex in conjunction with other techniques and did not report separate results specifically for ELISA or Luminex alone.

### Quality Assessment and Data Extraction

Two authors (SG and AE or ACP and JM) independently screened titles and abstracts to determine relevance for full text review. Two authors (SG and AE or LL and ACP) independently assessed full texts against the above inclusion and exclusion criteria and extracted data from included studies. Two authors (SG and LL) assessed the quality of included studies using The National Institutes of Health quality assessment tool for observational cohort and cross-sectional studies.^12^ Disagreements at any stage were resolved by discussion with a third author (CP).

Data was extracted on study demographics, CRS phenotypes, number of patients, tissue site of analysis, name and expression pattern of individual biomarkers.

### Outcomes and Data Synthesis

The primary outcome was the expression pattern of each biomarker in individual CRS phenotypes. A narrative synthesis of included studies was performed. Meta-analysis was not possible due to heterogeneity in assay techniques and study populations. Biomarkers were grouped by tissue site and CRS phenotype. Studies with conflicting results were identified separately.

## Results

### Characteristics of Included Studies

**Figure 1.**
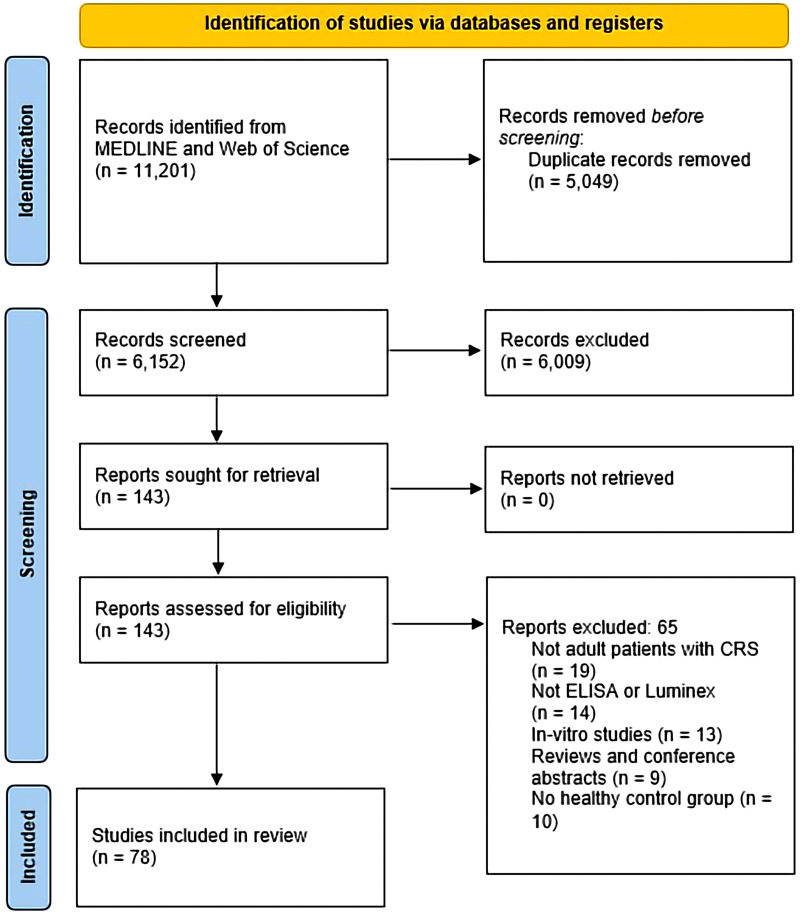
PRISMA 2020 flow diagram.^18^

Source: Page MJ, McKenzie JE, Bossuyt PM, Boutron I, Hoffmann TC, Mulrow CD, et al. The PRISMA 2020 statement: an updated guideline for reporting systematic reviews. BMJ 2021;372:n71. doi: 10.1136/bmj.n71.

**Figure 2.**
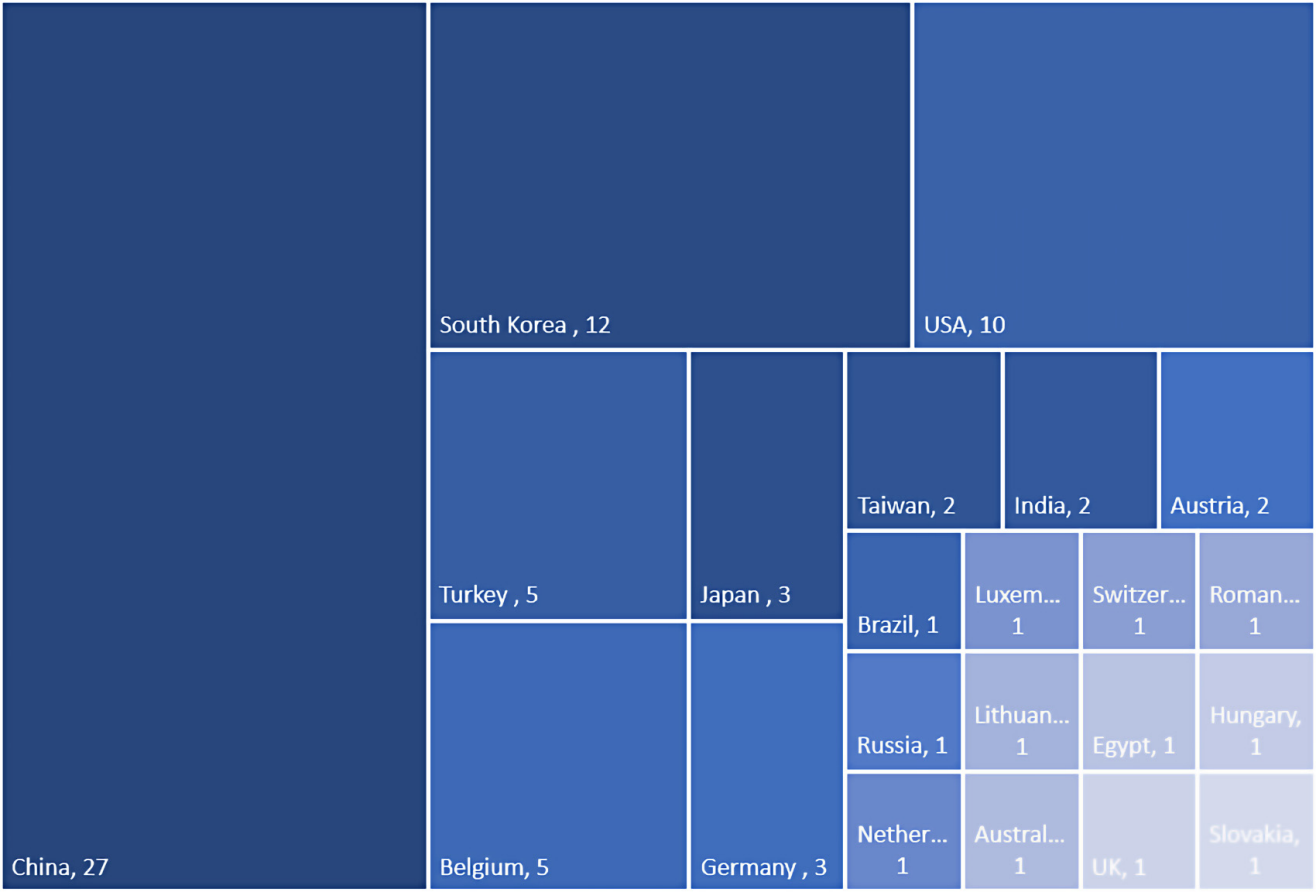
Geographical setting of included studies.

**Figure 3.**
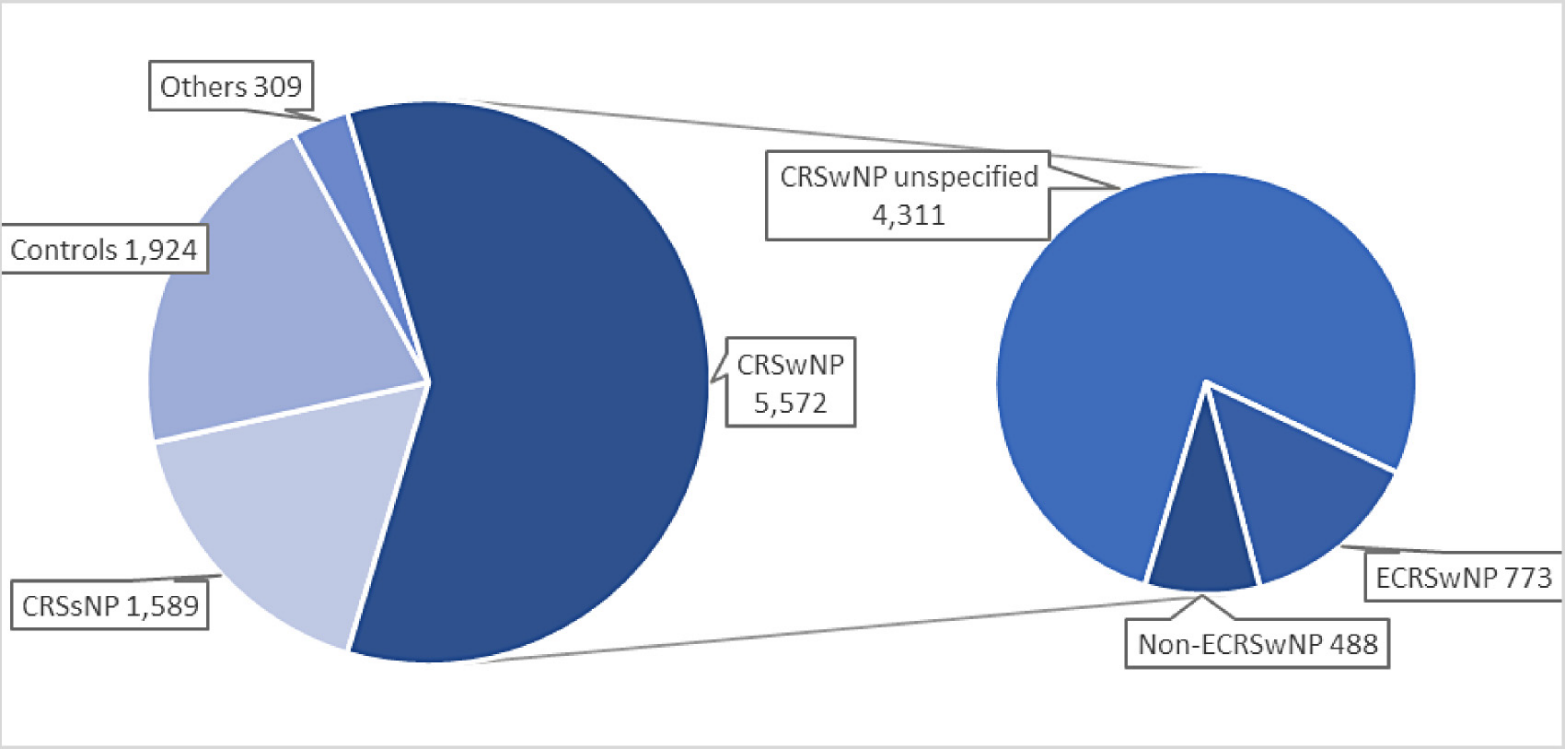
Patient phenotypes from included studies.

Abbreviations: CRSsNP, chronic rhinosinusitis without nasal polyposis; CRSwNP, chronic rhinosinusitis with nasal polyposis; ECRSwNP, eosinophilic chronic rhinosinusitis with nasal polyposis; non-ECRSwNP, non-eosinophilic chronic rhinosinusitis with nasal polyposis.

We identified 6152 unique records, of which 143 were selected for full text review (Figure 1). Seventy-eight studies met the inclusion criteria and were included in the final synthesis. Included studies were published between 2003^13^ and 2022.^14,15^ Twenty-seven studies were conducted in China, 12 in South Korea, 10 in the USA, five in Belgium and Turkey, three in Japan and Germany, two in Taiwan, India, and Austria, and one in Russia, Australia, the Netherlands, Luxembourg, Switzerland, Romania, Brazil, Egypt, Lithuania, Hungary, Slovakia, and the UK (Figure 2). The number of participants per study ranged from 20^16^ to 573.^17^ In total, up to 9394 participants were included across all 78 studies, of which up to 5572 patients had CRSwNP (Figure 3).

**Figure 4.**
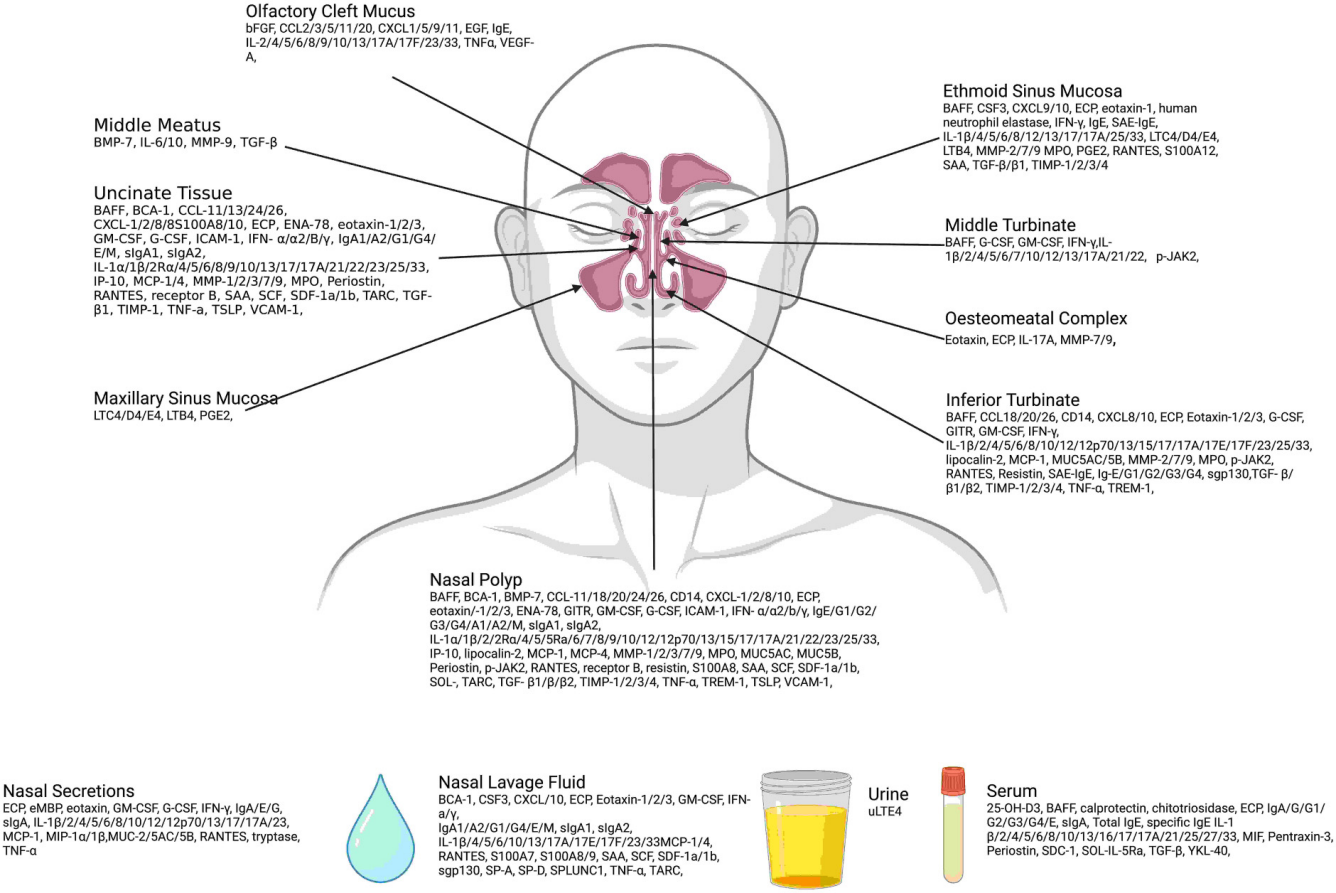
Site of identification of biomarkers. *Created with BioRender.com.*

A summary of biomarkers by site of identification is outlined in Figure 4. The majority of studies analyzed biomarkers from nasal tissue samples, including nasal polyps (44 studies), uncinate process (24 studies), inferior turbinate (21 studies), ethmoid mucosa (10 studies), middle turbinate (five studies), osteomeatal complex mucosa (two studies), maxillary sinus mucosa (one study), and unspecified nasal tissue (six studies). Five studies analyzed nasal secretions and a further seven studies analyzed nasal lavage fluid. Nineteen studies analyzed serum and one study analyzed urine.^19^ The most common phenotypic comparisons characterized by each biomarker are outlined in Table 2.

**Table 2.** Biomarkers Identified as Elevated or Decreased by CRS Phenotypic Comparison.

	Nasal tissue	Nasal secretions	Nasal lavage	Serum
CRSwNP to controls	↑**:** AREG, BAFF, ECP, ENA-78, eotaxin, eotaxin-1, eotaxin-2, eotaxin-3, G-CSF, GM-CSF, IFN-γ, LTC4/D4/E4, MCP-4, MMP-1, MMP-7, MMP-9, MPO, MUC5AC, MUC5B, pentraxin-3, p-JAK2, sgp130, TARC, TNF-α, TSLP ↓**:** PGE2, RANTES, TGF-β, TGF-β1	↑**:** ECP, eMBP, G-CSF, MCP-1, MIP-1α, MIP-1β, tryptase ↓**:** IFN-γ	↑**:** ECP, eotaxin-2, eotaxin-3, MCP-4	↑**:** calprotectin, chitotriosidase, ECP, MIF, pentraxin-3, periostin, SDC-1 ↓**:** 25-OH-D3
	↑**:** IL-1α, IL-1β, IL-1, IL-4, IL-5, IL-6, IL-8, IL-10^†^, IL-13, IL-17, IL-17A, IL-17rB, IL-21, IL-25, IL-33^†^, SOL-IL-5Ra ↓**:** IL-10^†^, IL-33^†^	↑**:** IL-1β, IL-4, IL-5, IL-8, IL-10^†^, IL-17 ↓**:** IL-10^†^, IL-12, IL-13	↑**:** IL-5, IL-13	↑**:** IL-1β, IL-5, IL-6, IL-8, IL-10, IL-13, IL-16, IL-17, IL-21, IL-25, IL-33, SOL-IL-5Ra ↓**:** IL-4
	↑**:** IgA1, sIgA2, IgE (total), IgG1-4, IgM			↑**:** IgE (total)
	↑**:** CCL-11, CCL-24, CXCL-1			
CRSsNP to controls	↑**:** ECP, human neutrophil elastase, ICAM-1, IFN-γ, MMP-1, MMP-7, MMP-9, MPO, S100A12, sgp130, TGF-β, TGF-β1, TNF-α ↓**:** 1,25-OH-D3	↑**:** MIP-1β, RANTES		↑**:** chitotriosidase, ECP, periostin ↓**:** 25-OH-D3
	↑**:** IL-1α, IL-1β, IL-4^†^, IL-5, IL-6, IL-8, IL-10^†^, IL-13, IL-17, IL-17A, IL-21, IL-22, IL-23, IL-25, IL-33 ↓**:** IL-4^†^, IL-10^†^	↑**:** IL-1β, IL-4, IL-5, IL-6, IL-8, IL-10 ↓**:** IL-13		↑**:** IL-1β, IL-5, IL-6, IL-8, IL-10, IL-21
	↑**:** IgE (total)			↑**:** IgE (total)
	↑**:** CCL-11, CCL-24, CXCL-1, CXCL-2			
CRSwNP to CRSsNP	↑**:** BAFF, ECP, ENA-78, eotaxin, eotaxin-1, eotaxin-2, LTC4/D4/E4, MCP-4, MPO, IFN-γ^†^, sgp130 ↓**:** human neutrophil elastase, IFN-γ^†^, PGE2, S100A12,TGF-β1, TIMP-1, TIMP-4, VEGF-A, 1,25-OH-D3	↓**:** VEGF-A	↑**:** eotaxin-2, eotaxin-3, MCP-4, TARC ↓**:** SP-A, SP-D	↑**:** MIF, periostin
	↑**:** IL-4, IL-5, IL-6, IL-9, IL-13, IL-17, IL-17A^†^, IL-25 ↓**:** IL-8, IL-17A^†^, IL-22, IL-33	↑**:** IL-4, IL-5, IL-9, IL-13 ↓**:** IL-2, IL-8	↑**:** IL-4, IL-13	
	↑**:** IgE (total), IgG1-4, SAE-IgE			
	↑**:** CCL2, CCL3, CCL-24, CXCL11 ↓**:** CXCL-2	↑**:** CCL2, CCL3, CXCL11		
ECRSwNP to controls	↑**:** ECP, eotaxin, eotaxin-2, eotaxin-3, GM-CSF, ICAM-1, IFN-β, IFN-γ, MCP-4, MMP-1, MMP-3, MMP-9, MCP-4, SAA, TSLP, VCAM-1			↑**:** BAFF, YKL-40
	↑**:** IL-1α, IL-1β, IL-2Rα, IL-4, IL-5, IL-6, IL-10, IL-12p70, IL-13, IL-15, IL-23, IL-25, IL-33			↑**:** IL-16
	↑**:** CCL-11, CCL-18, CCL-24, CCL26			
non-ECRSwNP to controls	↑**:** CD14, GITR, ICAM-1, IFN-γ, lipocalin-2, MCP-4, MMP-1, MMP-9, RANTES, resistin, SAA, TGF-β2, TREM-1, TSLP ↓**:** TIMP-1			↑**:** YKL-40
	↑**:** IL-1α, IL-1β, IL-2Rα, IL-4, IL-5, IL-6, IL-8, IL-13, IL-17A, IL-23, IL-25, IL-33			
	↑**:** CXCL-1, CXCL-2, CXCL-8, CCL-11, CCL-20, CCL-24			
ECRSwNP to non-ECRSwNP	↑**:** ECP, eotaxin, eotaxin-2, eotaxin-3, IFN-β, periostin, TSLP ↓**:** CD14, GITR, lipocalin-2, resistin, TGF-β2, TREM-1			↑**:** BAFF, MIF, YKL-40
	↑**:** IL-1β, IL-4, IL-5, IL-13, IL-10, IL-12p70, IL-13, IL-15, IL-25, IL-33 ↓**:** IL-8, IL-17A, IL-17			
	↑**:** CCL-11, CCL18 ↓**:** CCL-20			

↑ = elevated in phenotypic comparison.

↓ = decreased in phenotypic comparison.

† = studies showed conflicting results.

Abbreviations: 1,25-OH-D3, calcitriol; 25-OH-D3, calcifediol; AERD, aspirin-exacerbated respiratory disease; AREG, amphiregulin; *A. flavus, Aspergillus flavus*; AFRS, allergic fungal rhinosinusitis; BAFF, B-cell activating factor; BCA, B cell-attracting chemokine; bFGF, basic fibroblast growth factor; BMP, bone morphogenetic protein; CCL, C-C motif chemokine ligand; CD, cluster of differentiation; CRS, chronic rhinosinusitis; CRSsNP, chronic rhinosinusitis without nasal polyposis; CRSwNP, chronic rhinosinusitis with nasal polyposis; CSF, colony stimulating factor; CT, computed tomography; CXCL, C-X-C motif chemokine ligand; ECP, eosinophilic cationic protein; ECRS, eosinophilic chronic rhinosinusitis; ECRSwNP, eosinophilic chronic rhinosinusitis with nasal polyposis; EGF, epidermal growth factor; eMBP, eosinophil major basic protein; ENA, extractable nuclear antigen; G-CSF, granulocyte colony stimulating factor; GITR, glucocorticoid induced tumor necrosis factor related protein; GM-CSF, granulocyte macrophage colony stimulating factor; ICAM, Intercellular adhesion molecule; IFN-γ, interferon gamma; IFN-β, interferon beta; Ig, immunoglobulin; IL, interleukin; IL-2Rα, interleukin 2 subunit receptor alpha; IP, Interferon gamma-induced protein; LMK-CT, Lund–Mackay computed tomography; LT, leukotriene; MCP, monocyte chemoattractant protein; MIF, macrophage migration inhibitory factor; MIP, macrophage inflammatory protein; MMP, matrix metalloproteinase; MPO, myeloperoxidase; MUC, mucin; NERD, non-steroidal anti-inflammatory drug-exacerbated respiratory disease; Non-ECRS, non-eosinophilic chronic rhinosinusitis; non-ECRSwNP, non-eosinophilic chronic rhinosinusitis with nasal polyposis; non-T1, non-type 1 inflammation; PGE2, prostaglandin E2; p-JAK, phosphorylated janus activating kinase; QoL, quality of life; RANTES, regulated upon activation, normal T cell expressed and presumably secreted (CCL5), S100A12—S100 calcium-binding protein A12; S100A7, calcium binding protein A7; S100A8, calcium binding protein A8; SAA, serum amyloid A; SAE-IgE, immunoglobulin E antibodies to *Staphylococcus aureus* enterotoxin; SCF, stem cell factor; SDF, stromal cell derived factor; SDC-1, syndecan 1; sgp130, soluble glycoprotein 130; sIgA, secretory immunoglobulin A; SNOT-22–22 item sinonasal outcome test; SOL-IL-5Ra, soluble interleukin 5 receptor alpha; SP, surfactant protein; SPLUNC1, short-palate lung and nasal epithelial clone 1; T1, type 1 inflammation; T3, type 3 inflammation; TARC, thymus and activation-regulated chemokine; TGF-β, transforming growth factor beta; TIMP, tissue inhibitor of metalloproteinases; TNF-a, tumor necrosis factor alpha; TREM, triggering receptor expressed on myeloid cells; TSLP, thymic stromal lymphopoietin; uLTE4, urinary leukotriene E4; VCAM, vascular cellular adhesion molecule; VEGF, vascular endothelial growth factor; YKL-40, chitinase-3-like protein 1.

### Quality Assessment

The quality assessment scores for individual studies are outlined in Supplemental file 2. Twenty-five studies received an overall quality rating of “good,” 43 as “fair,” and 10 as “poor.” Most studies had clearly defined outcome measures but only eight studies commented on blinding of participants and only two provided a sample size justification. All included studies were of cross-sectional or cohort study design.

### Serum Biomarkers

Twenty-four biomarkers were identified in serum samples of CRS patients from 19 studies (Table 3). The majority were found to distinguish patients with CRSwNP or CRSsNP from controls, and only four biomarkers were found to differentiate CRS phenotypes. Macrophage migration inhibitory factor and periostin were elevated in CRSwNP compared to CRSsNP.^20,21^ Chitinase-3-like protein 1 (YKL-40) and B-cell activating factor (BAFF) were found to distinguish patients with ECRSwNP and recurrent CRSwNP.^14,22^

**Table 3.** Studies of Serum Biomarkers.

Study Authors	Year/country	Patients	Biomarkers	Expression pattern
van Zele et al^25^	2007 Belgium	15 CRSwNP, 15 CRSsNP, 10 controls	IgG1, IgG2, IgG3, IgG4	No difference between CRSwNP patients compared to CRSsNP and controls.
Lackner et al^16^	2007 Austria	10 ECRS 10 controls	IL-16	IL-16 increased in ECRS compared to controls.
Gevaert et al^26^	2009 Germany	34 CRSwNP, 16 controls	SOL-IL-5Ra, IL-5	SOL-IL-5Ra and IL-5 increased in CRSwNP compared to controls.
Keseroglu et al^27^	2012 Turkey	17 CRSwNP, 10 controls	IL-16	IL-16 increased in CRSwNP compared to controls.
Chao et al^28^	2015 Taiwan	37 CRSwNP, 18 CRSsNP, 37 controls	IL-21, IgE	IL-21 and IgE were increased in CRSwNP and CRSsNP compared with controls. No difference between CRS groups.
Cui et al^29^	2015 China	40 CRSwNP, 30 CRSsNP, 30 controls	Total IgE, specific IgE and ECP	Total IgE and ECP were increased in CRSwNP and CRSsNP compared to controls. No difference between CRS groups.
Tsybikov et al ^30^	2015 Russia	54 CRSwNP, 46 CRSsNP, 40 controls	IgA, IgE, sIgA, IgG, IL-1B, IL-2, IL-4, IL-5, IL-6, IL-8, IL-10, and IL17A	IL-1B, IL-5, IL-6, IL-8, and IL-10 were increased in CRSwNP and CRSsNP compared with controls. No difference between CRS and controls for IL-2, IL-4, and IL-17A.
Qin et al^31^	2016 China	25 CRSwNP, 12 CRSsNP, 15 controls	Periostin	Increased in CRSwNP and CRSsNP compared with controls.
Ozturan et al ^32^	2017 Turkey	20 CRSwNP 20 CRSsNP 20 controls	IL-25 and IL-33	Il-25 and IL-33 did not differ between CRS groups and controls.
Maxfield et al ^20^	2018 USA	33 CRSwNP 38 CRSsNP 62 controls	Periostin	Increased in CRS groups compared with controls. Increased in CRSwNP compared to CRSsNP.
Rai et al^33^	2018 India	31 CRSwNP with *A. flavus* infection 20 controls	IL-1β, IL-2, IL-4, IL-6, IL-17, IL-21, IL-27, TGF-β, and total IgE	IL-1β, IL-17, IL-21, TGF-β, and total IgE were increased in CRSwNP with *A. flavus* infection compared to controls. IL-2, IL-4, IL-6, and IL-27 were decreased in CRSwNP with *A. flavus* infection compared to controls.
Candar et al^24^	2020 Turkey	26 CRSwNP 24 CRSsNP 27 controls	calprotectin	Calprotectin was increased in CRSwNP compared with controls. Calprotectin was increased in CRSwNP with AERD compared with CRSwNP without AERD.
Dutu et al^34^	2020 Romania	11 CRSwNP 5 CRSsNP 21 controls	chitotriosidase, 25-OH-D3	Chitotriosidase was increased in CRSwNP and CRSsNP compared with control. 25-OH-D3 was decreased in CRSwNP and CRSsNP compared with controls.
Gulluev et al^35^	2020 Turkey	35 CRSwNP 29 controls	IL-13, Il-25, IL-33, SDC-1	IL13, IL-25, IL-33, SDC-1 increased in CRSwNP compared to controls.
Yuan et al^21^	2021 China	51 ECRSwNP, 69 non-ECRSwNP 40 CRSsNP 40 controls	MIF	MIF was increased in CRSwNP compared with CRSsNP and controls. MIF was increased in ECRSwNP compared with non-ECRSwNP.
Wen et al^22^	2021 China	37 primary ECRSwNP 43 primary non-ECRSwNP 40 recurrent CRSwNP 40 controls	YKL-40	YKL-40 was increased in ECRSwNP and non-ECRSwNP compared with controls. YKL-40 was increased in ECRSwNP compared with non-ECRSwNP. YKL-40 was increased in recurrent CRSwNP compared with primary ECRSwNP.
Hussien et al ^23^	2021 Egypt	50 CRSwNP 25 controls	IgE, IL-17, Pentraxin-3	IgE, IL-17, and Pentraxin-3 were increased in CRSwNP compared with controls and were correlated with SNOT-22 and LMK-CT scores.
Wang et al^14^	2022 China	52 ECRSwNP, 68 non-ECRSwNP, 60 controls	BAFF	BAFF was increased in ECRSwNP compared with non-ECRSwNP and controls. BAFF was increased in recurrent CRSwNP compared with non-recurrent CRSwNP. BAFF was increased in recurrent ECRSwNP compared with non-recurrent ECRSwNP and with recurrent and non-recurrent non-ECRSwNP.
Shrestha et al ^15^	2022 India	30 primary CRSwNP 30 recurrent CRSwNP 30 controls	IL-4, IL-5, IL-13	IL-5 and IL-13 were increased in primary and recurrent CRSwNP compared with control. IL-4 was decreased in primary and recurrent CRSwNP compared with controls.

.

Abbreviations: 1,25-OH-D3, calcitriol; 25-OH-D3, calcifediol; AERD, aspirin-exacerbated respiratory disease; AREG, amphiregulin; *A. flavus*, *Aspergillus flavus*; AFRS, allergic fungal rhinosinusitis; BAFF, B-cell activating factor; BCA, B cell-attracting chemokine; bFGF, basic fibroblast growth factor; BMP, bone morphogenetic protein; CCL, C-C motif chemokine ligand; CD, cluster of differentiation; CRS, chronic rhinosinusitis; CRSsNP, chronic rhinosinusitis without nasal polyposis; CRSwNP, chronic rhinosinusitis with nasal polyposis; CSF, colony stimulating factor; CT, computed tomography; CXCL, C-X-C motif chemokine ligand; ECP, eosinophilic cationic protein; ECRS, eosinophilic chronic rhinosinusitis; ECRSwNP, eosinophilic chronic rhinosinusitis with nasal polyposis; EGF, epidermal growth factor; eMBP, eosinophil major basic protein; ENA, extractable nuclear antigen; G-CSF, granulocyte colony stimulating factor; GITR, glucocorticoid induced tumor necrosis factor related protein; GM-CSF, granulocyte macrophage colony stimulating factor; ICAM, Intercellular adhesion molecule; IFN-γ, interferon gamma; IFN-β, interferon beta; Ig, immunoglobulin; IL, interleukin; IL-2Rα, interleukin 2 subunit receptor alpha; IP, Interferon gamma-induced protein; LMK-CT, Lund–Mackay computed tomography; LT, leukotriene; MCP, monocyte chemoattractant protein; MIF, macrophage migration inhibitory factor; MIP, macrophage inflammatory protein; MMP, matrix metalloproteinase; MPO, myeloperoxidase; MUC, mucin; NERD, non-steroidal anti-inflammatory drug-exacerbated respiratory disease; Non-ECRS, non-eosinophilic chronic rhinosinusitis; non-ECRSwNP, non-eosinophilic chronic rhinosinusitis with nasal polyposis; non-T1, non-type 1 inflammation, PGE2, prostaglandin E2; p-JAK, phosphorylated janus activating kinase; QoL, quality of life; RANTES, regulated upon activation, normal T cell expressed and presumably secreted (CCL5); S100A12, S100 calcium-binding protein A12; S100A7, calcium binding protein A7; S100A8, calcium binding protein A8; SAA, serum amyloid A; SAE-IgE, immunoglobulin E antibodies to *Staphylococcus aureus* enterotoxin; SCF, stem cell factor; SDF, stromal cell derived factor; SDC-1, syndecan 1; sgp130, soluble glycoprotein 130; sIgA, secretory immunoglobulin A; SNOT-22–22 item sinonasal outcome test; SOL-IL-5Ra, soluble interleukin 5 receptor alpha; SP, surfactant protein; SPLUNC1, short-palate lung and nasal epithelial clone 1; T1, type 1 inflammation; T3, type 3 inflammation; TARC, thymus and activation-regulated chemokine; TGF-β, transforming growth factor beta; TIMP, tissue inhibitor of metalloproteinases; TNF-a, tumor necrosis factor alpha; TREM, triggering receptor expressed on myeloid cells; TSLP, thymic stromal lymphopoietin; uLTE4, urinary leukotriene E4; VCAM, vascular cellular adhesion molecule; VEGF, vascular endothelial growth factor, YKL-40, chitinase-3-like protein 1.

Studies of serum biomarkers for CRS disease activity were limited. However, a study of 50 patients with CRSwNP by Hussein et al found that serum immunoglobulin E (IgE), interleukin-17 (IL-17), and pentraxin-3 were correlated with SNOT-22 and LMK-CT scores.^23^ Study quality was fair due to the use of age and sex matched controls but no specific CRS inclusion criteria. Candar et al studied 26 patients with CRSwNP and found that serum calprotectin was elevated in those with aspirin-exacerbated respiratory disease (AERD) compared to those without AERD.^24^ Study quality was limited by a small sample size of six AERD patients.

### Nasal Secretion Biomarkers

Twenty-five biomarkers were identified in nasal secretions across five studies (Table 4). Nasal secretion harvesting techniques differed in each study. Secretions were sampled using cotton swabs applied to the inferior turbinate,^36^ cotton swabs with phosphate-buffered solution in the anterior nares,^30^ absorbent cotton wool left for 20 min in the middle meatus,^37^ neurosurgical patties left above the inferior turbinate for 10 min,^38^ or using a “Sinus Secretion Collector” in the middle meatus.^39^

**Table 4.** Studies of Nasal Secretion Biomarkers.

Study authors	Year/country	Patients	Biomarkers	Expression pattern
Ali et al^36^	2005 UK	11 CRSwNP, 8 CRSsNP, 10 controls	MUC2, MUC5AC, MUC5B	No difference between CRSwNP and CRSsNP.
Schmid et al^39^	2010 Austria	23 CRSwNP, 21 controls	eMBP	eMBP increased in CRSwNP compared to controls.
Tsybikov et al ^30^	2015 Russia	54 CRSwNP, 46 CRSsNP, 40 controls	IgA, IgE, sIgA, IgG, IL-1B, IL-2, IL-4, IL-5, IL-6, IL-8, IL-10, and IL17A	IL-1B, IL-5, IL-6, IL-8, and IL-10 were increased in CRSwNP and CRSsNP compared with controls. IL-2 increased in CRSsNP compared with CRSwNP. IL-4, and IL-5 increased in CRSwNP compared to CRSsNP. No difference between CRS groups.
Konig et al^37^	2016 Germany	45 CRSwNP, 48 CRSsNP, 48 controls	IL-4, IL-5, IL-10, IL-12, IL-13, IL-17, IL-8, GM-CSF, G-CSF, IFN-γ, MCP-1, MIP-1α, MIP-1β, eotaxin, RANTES, ECP, and tryptase,	IL-5, IL-17, G-CSF, MCP-1, MIP-1α, MIP-1β, ECP, and tryptase were increased in CRSwNP compared to controls while IL-10, IL-12, IL-13, and IFN-γ were decreased. RANTES and MIP-1β were increased in CRSsNP compared to controls while IL-13 was decreased. IL-4, IL-8, GM-CSF, and eotaxin showed no difference between the three groups.
Steiner et al ^38^	2020 Switzerland	13 CRSwNP with NERD 13 CRSwNP without NERD 15 controls	Tryptase, IL-4, IL-5, IL-6, IL-8, IL-12p70, IL-13, IL-17A, IL-23, IFN-γ, TNF-a	IL-6 and IL-5 were increased in CRSwNP without NERD compared to controls. IL-5 and IL-13 were increased in CRSwNP with NERD compared to CRSwNP without NERD. Tryptase and IL-13 were increased in CRSwNP with NERD compared to controls.

Abbreviations: 1,25-OH-D3, calcitriol; 25-OH-D3, calcifediol; AERD, aspirin-exacerbated respiratory disease; AREG, amphiregulin; *A. flavus*, *Aspergillus flavus*; AFRS, allergic fungal rhinosinusitis; BAFF, B-cell activating factor; BCA, B cell-attracting chemokine; bFGF, basic fibroblast growth factor; BMP, bone morphogenetic protein; CCL, C-C motif chemokine ligand; CD, cluster of differentiation; CRS, chronic rhinosinusitis; CRSsNP, chronic rhinosinusitis without nasal polyposis; CRSwNP, chronic rhinosinusitis with nasal polyposis; CSF, colony stimulating factor; CT, computed tomography; CXCL, C-X-C motif chemokine ligand; ECP, eosinophilic cationic protein; ECRS, eosinophilic chronic rhinosinusitis; ECRSwNP, eosinophilic chronic rhinosinusitis with nasal polyposis; EGF, epidermal growth factor; eMBP, eosinophil major basic protein; ENA, extractable nuclear antigen; G-CSF, granulocyte colony stimulating factor; GITR, glucocorticoid induced tumor necrosis factor related protein, GM-CSF, granulocyte macrophage colony stimulating factor; ICAM, intercellular adhesion molecule; IFN-γ, interferon gamma; IFN-β, interferon beta; Ig, immunoglobulin; IL, interleukin; IL-2Rα, interleukin 2 subunit receptor alpha; IP, Interferon gamma-induced protein; LMK-CT, Lund–Mackay computed tomography; LT, leukotriene; MCP, monocyte chemoattractant protein; MIF, macrophage migration inhibitory factor; MIP, macrophage inflammatory protein; MMP, matrix metalloproteinase; MPO, myeloperoxidase; MUC, mucin; NERD, non-steroidal anti-inflammatory drug-exacerbated respiratory disease; Non-ECRS, non-eosinophilic chronic rhinosinusitis; non-ECRSwNP, non-eosinophilic chronic rhinosinusitis with nasal polyposis; non-T1, non-type 1 inflammation; PGE2, prostaglandin E2; p-JAK, phosphorylated janus activating kinase; QoL, quality of life; RANTES, regulated upon activation; normal T cell expressed and presumably secreted (CCL5); S100A12, S100 calcium-binding protein A12; S100A7, calcium binding protein A7; S100A8, calcium binding protein A8; SAA, serum amyloid A; SAE-IgE, immunoglobulin E antibodies to *Staphylococcus aureus* enterotoxin; SCF, stem cell factor; SDF, stromal cell derived factor; SDC-1, syndecan 1; sgp130, soluble glycoprotein 130; sIgA, secretory immunoglobulin A; SNOT-22–22 item sinonasal outcome test; SOL-IL-5Ra, soluble interleukin 5 receptor alpha; SP, surfactant protein; SPLUNC1, short-palate lung and nasal epithelial clone 1; T1, type 1 inflammation; T3, type 3 inflammation; TARC, thymus and activation-regulated chemokine; TGF-β, transforming growth factor beta; TIMP, tissue inhibitor of metalloproteinases; TNF-a, tumor necrosis factor alpha; TREM, triggering receptor expressed on myeloid cells; TSLP, thymic stromal lymphopoietin; uLTE4, urinary leukotriene E4; VCAM, vascular cellular adhesion molecule; VEGF, vascular endothelial growth factor; YKL-40, chitinase-3-like protein 1.

Biomarkers found to differentiate CRS phenotypes included IL-4, IL-5, IL-9, IL-13, C-C motif chemokine ligand 2 (CCL2), CCL3, C-X-C motif chemokine ligand 11 (CXCL11), and IgE which were increased in CRSwNP compared to CRSsNP, while the reverse was true for IL-2, IL-8, and vascular endothelial growth factor A (VEGF-A). Immunoglobulins were not associated with CRS phenotypes in nasal secretions.

Steiner et al studied 13 patients with CRSwNP and non-steroidal anti-inflammatory drug-exacerbated respiratory disease (NERD) and 13 patients with CRSwNP without NERD. They found that the former group had increased levels of IL-5 and IL-13.^38^ Study quality was limited by a small sample size of 13 NERD patients and no specific CRSwNP inclusion criteria.

### Nasal Lavage Fluid Biomarkers

Fourteen biomarkers were identified in nasal lavage fluid across seven studies (Table 5). IL-4, IL-13, eotaxin-2, eotaxin-3, monocyte chemoattractant protein-4, and thymus and activation-regulated chemokine were increased in CRSwNP compared to CRSsNP. No studies of CRSwNP subtypes were performed and immunoglobulins were not found to distinguish CRS phenotypes. The largest study was by Klingler et al of 126 patients with CRSsNP which identified that CXCL9 and CXCL10 were elevated in patients characterized by the T1 endotype compared to non-T1 CRSsNP and controls.^40^ Study quality was fair with detailed CRS inclusion criteria but nasal lavage fluid samples were highly diluted.

**Table 5.** Studies of Nasal Lavage Biomarkers.

Study Authors	Year/country	Patients	Biomarkers	Expression pattern
Peters et al^41^	2010 USA	38 CRSwNP, 30 CRSsNP, 18 controls	IL-6, sIL-6R, sgp130	No difference between groups.
Hulse et al^42^	2013 USA	16 CRSwNP, 15 CRSsNP, 17 controls	IgG1, IgG4, IgE, IgA1, IgA2, IgM, sIgA1, sIgA2	IgG1, IgG4, IgE, IgM, IgA1, and sIgA2 increased in CRSwNP compared to controls.
Uhliarova et al^43^	2015 Slovakia	31 CRSwNP, 13 CRSsNP, 17 controls	SP-A, SP-D	SP-A and SP-D increased in CRSsNP compared to CRSwNP and controls.
Stevens et al ^44^	2015 USA	30 CRSwNP, 9 CRSsNP, 17 controls	ECP, IL-4, IL-5, IL-6, IL-10, IL-13, IL-33, eotaxin-1, eotaxin-2, eotaxin-3, RANTES, MCP-4, TARC, SCF, GM-CSF, MCP-1, IFN-a2, IFN-γ, BCA-1, SDF-1a, and SDF-1b	ECP, IL-5, IL-13, eotaxin-2, eotaxin-3, and MCP-4 were increased in CRSwNP compared with controls. IL-4, IL-13, eotaxin-2, eotaxin-3, MCP-4, and TARC were increased in CRSwNP compared with CRSsNP.
Min et al^45^	2017 USA	51 CRSwNP, 18 CRSsNP, 7 controls	IL-4, IL-13, Eotaxin-1, Eotaxin-2, Eotaxin-3	IL-13, Eotaxin-2, and Eotaxin-3 increased in CRSwNP compared to controls.
Kim et al^46^	2019 Korea	45 CRS with fungal balls, 6 CRS with bacterial balls, 27 CRS with mixed balls, 2 CRS with double balls, 10 controls	TNF-α, IL-1β, S100A7, S100A8/9, SPLUNC1	TNF-α was increased in CRS with fungal and mixed balls compared to controls. IL-1B increased in CRS with mixed balls compared to CRS with fungal balls. S100A7 and S100A8/A9 increased in CRS with fungal balls compared to CRS with mixed balls. No difference in SPLUNC1 expression.
Klingler et al ^40^	2021 USA	55 CRSwNP 126 CRSsNP 42 controls	CXCL9, CXCL10, CSF3, SAA, IL-1β, IL-6	CXCL9 and CXCL10 were increased in T1 CRSsNP compared with non-T1 CRSsNP and controls. CSF3 was increased in T3 CRSsNP compared with controls. SAA, IL-1β, and IL-6 showed no difference between T3 CRSsNP and controls

Abbreviations: 1,25-OH-D3, calcitriol, 25-OH-D3; calcifediol; AERD, aspirin-exacerbated respiratory disease; AREG, amphiregulin; *A. flavus*, *Aspergillus flavus*; AFRS, allergic fungal rhinosinusitis; BAFF, B-cell activating factor; BCA, B cell-attracting chemokine; bFGF, basic fibroblast growth factor; BMP, bone morphogenetic protein; CCL, C-C motif chemokine ligand; CD, cluster of differentiation; CRS, chronic rhinosinusitis; CRSsNP, chronic rhinosinusitis without nasal polyposis; CRSwNP, chronic rhinosinusitis with nasal polyposis; CSF, colony stimulating factor; CT, computed tomography; CXCL, C-X-C motif chemokine ligand; ECP, eosinophilic cationic protein; ECRS, eosinophilic chronic rhinosinusitis; ECRSwNP, eosinophilic chronic rhinosinusitis with nasal polyposis; EGF, epidermal growth factor; eMBP, eosinophil major basic protein; ENA, extractable nuclear antigen; G-CSF, granulocyte colony stimulating factor; GITR, glucocorticoid induced tumor necrosis factor related protein; GM-CSF, granulocyte macrophage colony stimulating factor; ICAM, Intercellular adhesion molecule; IFN-γ, interferon gamma; IFN-β, interferon beta; Ig, immunoglobulin; IL, interleukin; IL-2Rα, interleukin 2 subunit receptor alpha; IP, Interferon gamma-induced protein; LMK-CT, Lund–Mackay computed tomography; LT, leukotriene; MCP, monocyte chemoattractant protein; MIF, macrophage migration inhibitory factor; MIP, macrophage inflammatory protein; MMP, matrix metalloproteinase; MPO, myeloperoxidase; MUC, mucin; NERD, non-steroidal anti-inflammatory drug-exacerbated respiratory disease; Non-ECRS, non-eosinophilic chronic rhinosinusitis; non-ECRSwNP, non-eosinophilic chronic rhinosinusitis with nasal polyposis; non-T1, non-type 1 inflammation; PGE2, prostaglandin E2; p-JAK, phosphorylated janus activating kinase; QoL, quality of life; RANTES, regulated upon activation, normal T cell expressed and presumably secreted (CCL5); S100A12, S100 calcium-binding protein A12; S100A7, calcium binding protein A7; S100A8, calcium binding protein A8; SAA, serum amyloid A; SAE-IgE, immunoglobulin E antibodies to *Staphylococcus aureus* enterotoxin; SCF, stem cell factor; SDF, stromal cell derived factor; SDC-1, syndecan 1; sgp130, soluble glycoprotein 130; sIgA, secretory immunoglobulin A; SNOT-22, 22-item sinonasal outcome test; SOL-IL-5Ra, soluble interleukin 5 receptor alpha; SP, surfactant protein; SPLUNC1, short-palate lung and nasal epithelial clone 1; T1, type 1 inflammation; T3, type 3 inflammation; TARC, thymus and activation-regulated chemokine; TGF-β, transforming growth factor beta; TIMP, tissue inhibitor of metalloproteinases; TNF-a, tumor necrosis factor alpha; TREM, triggering receptor expressed on myeloid cells; TSLP, thymic stromal lymphopoietin; uLTE4, urinary leukotriene E4; VCAM, vascular cellular adhesion molecule; VEGF, vascular endothelial growth factor; YKL-40, chitinase-3-like protein 1.

### Nasal Tissue Biomarkers

Eighty biomarkers across 55 studies (Table 6) were identified in nasal tissue, including 57 differentiating CRSwNP from controls, 35 differentiating CRSsNP from controls, 41 differentiating CRSwNP from CRSsNP, 30 differentiating ECRSwNP from non-ECRSwNP, and 12 differentiating refractory CRSwNP from controls or primary CRSwNP. The most widely measured biomarker among included studies was IL-5, followed by IL-13 and IL-4.

**Table 6.** Studies of Nasal Tissue Biomarkers.

Study Authors	Year/country	Patients	Tissue	Biomarkers	Expression pattern
Hirschberg et al^13^	2003 Hungary	34 CRSwNP 9 controls	Nasal polyp, inferior turbinate	IL-5, TGF-B1, IgE	IL-5 and tissue IgE increased in CRSwNP compared to controls. TGF-B1 increased in controls compared to CRSwNP.
Perez-Novo et al^60^	2006 Belgium	13 CRSwNP, 11 CRSsNP, 6 controls	Ethmoid and maxillary sinus mucosa	PGE2, LTC4/D4/E4, LTB4	PGE2 increased in CRSsNP and controls compared to CRSwNP. LTC4/D4/E4 increased in CRSwNP compared to CRSsNP and controls. No difference in LTB4 between groups.
van Zele et al^25^	2007 Belgium	15 CRSwNP, 15 CRSsNP, 10 controls	Nasal polyp, inferior turbinate	IgG1, IgG2, IgG3, IgG4	Increased in nasal mucosa in CRSwNP patients compared to CRSsNP and controls.
Liu et al^49^	2009 China	20 CRSwNP, 17 CRSsNP, 12 controls	Nasal polyp, sinonasal mucosa	TNF-a, IL-1β, IL-4, IFN-γ, and IL-10	TNF-a, IL-1β, and IL-10 increased in CRSwNP and CRSsNP compared to controls. IFN-γ increased in CRSsNP compared to controls. IL-4 did not differ between groups.
Gevaert et al^26^	2009 Germany	34 CRSwNP, 16 controls	Nasal polyp	SOL-IL-5Ra, IL-5	SOL-IL-5Ra and IL-5 increased in CRSwNP compared to controls.
Shi et. al.^61^	2009 China	24 CRSwNP, 11 controls	Nasal polyp	IL-4, IL-5, IFNy, IL-10, IL-17, TGFB	IFNy, IL-4, IL-5 increased in CRSwNP compared to controls. IL-10 and TGFB increased in controls compared to CRSwNP.
Li et al^62^	2010 China	12 CRSwNP, 12 CRSsNP, 12 controls	Nasal polyp, ethmoid mucosa, inferior turbinate	MMP-2, MMP-7, MMP-9, TIMP-1, TIMP-2, TIMP-3, TIMP-4, and TGF-β1	TGF-β1, TIMP-1, TIMP-4 increased in CRSsNP compared to CRSwNP. MMP-7 and 9 increased in CRSwNP and CRSsNP compared to controls. MMP-2, TIMP-2, and TIMP-3 did not differ between groups.
Peters et al^41^	2010 USA	38 CRSwNP, 30 CRSsNP, 18 controls	Nasal tissue, inferior turbinate	IL-6, IL-17A, IL-17-E, IL-17F, IL-23, and sgp130	IL-6 and sgp130 were increased in CRSwNP compared to CRSsNP and controls. IL-17A, IL-17E, IL-17F, and IL-23 undetectable.
Sejima et al^63^	2012 Japan	19 CRSwNP, 9 CRSsNP, 14 controls	Ethmoid mucosa, inferior turbinate	TGF-β, IL-5, IgE, SAE-IgE, ECP, MPO, IL-1β, IL-6, and IL-8	IL-5, IgE, SAE-IgE, and ECP were increased in CRSwNP compared to CRSsNP. ECP/MPO ratio and IL-8 increased in CRSwNP compared to controls. TGF-β, MPO, IL-6, and IL-1β increased in CRSsNP compared to controls.
Hulse et al^42^	2013 USA	16 CRSwNP, 15 CRSsNP, 17 controls	Uncinate process, nasal polyp	IgG1, IgG4, IgE, IgA1, IgA2, IgM, sIgA1, sIgA2	IgG1, IgG4, IgE, IgM, IgA1, and sIgA2 increased in CRSwNP compared to controls.
Derycke et al^64^	2014 Belgium	15 CRSwNP, 9 CRSsNP, 7 controls	Sinonasal mucosa, inferior turbinate	IL-4, IL-5, IL-6, IL-8, IL-17, IL-1β, IFN-γ, IgE, and ECP	IL-4, IL-5, and IgE were increased in CRSwNP compared to controls. IL-1b, IL-6, IL-8, IL-17, and IFN-γ showed no difference between groups.
Li et al^65^	2014 China	41 CRSwNP, 20 CRSsNP, 19 controls	Nasal polyp, ethmoid mucosa, uncinate process	IL-5, IL-6, and IL-8	IL-5 and IL-8 were increased in CRSwNP and CRSsNP compared to controls. IL-6 was increased in CRSwNP compared to controls
Xiao et al^66^	2014 China	64 CRSwNP, 25 CRSsNP, 29 controls	Nasal polyp, uncinate process	IL-21	Increased in CRSwNP and CRSsNP compared to controls. IL-21 levels were associated with polyp size and recurrence after surgery.
Shin et al^67^	2015 South Korea	122 CRSwNP, 65 CRSsNP, 27 controls	Nasal polyp, uncinate process,	IL-25 and IL-17 receptor B	IL-25 increased in CRSwNP compared with CRSsNP and controls. IL-17 receptor B increased in CRSwNP compared to controls.
Stevens et al ^44^	2015 USA	30 CRSwNP, 9 CRSsNP, 17 controls	Nasal polyp, uncinate process	ECP, IL-4, IL-5, IL-6, IL-10, IL-13, IL-33, eotaxin-1, eotaxin-2, eotaxin-3, RANTES, MCP-4, TARC, SCF, GM-CSF, MCP-1, IFN-a2, IFN-γ, BCA-1, SDF-1a, and SDF-1b	ECP, IL-5, IL-10, IL-13, Eotaxin-1, Eotaxin-2, Eotaxin-3, MCP-4, and TARC were increased in CRSwNP compared to controls. IL-4 increased in controls compared to CRSsNP. RANTES increased in controls compared to CRSwNP. ECP, IL-4, IL-5, IL-13, Eotaxin-2, and MCP-4 increased in CRSwNP compared to CRSsNP. SDF-1, SCF, GM-CSF, BCA-1, IFN-γ, IFN-a, and IL-33 did not differ between CRS groups.
Schlosser et al^68^	2015 USA	13 CRSwNP, 13 CRSsNP, 6 AFRS, 18 controls	Nasal tissue	25-OH-D3, 1,25-OH-D3	1,25-OH-D3 increased in controls and CRSsNP compared to CRSwNP and AFRS.
Kim et al^51^	2016 South Korea	140 CRSwNP, 61 CRSsNP, 19 controls	Uncinate process, nasal polyp	IL-33	Increased in CRSwNP and CRSsNP compared with controls.
Wang et al^17^	2016 Australia, Belgium, Luxembourg, Netherlands, Japan, Germany	271 CRSwNP, 164 CRSsNP, 138 controls	Nasal polyp, ethmoid mucosa, inferior turbinate	IL-5, IFN-γ, IL-17, IL-8, TGF-β1	IL-8 was increased in CRSwNP compared to controls in all regions. IL-5 was increased in CRSwNP compared to CRSsNP and controls from Benelux, Berlin, Adelaide, Beijing, and Tochigi, but not Chengdu. IL-17 was increased in CRSwNP compared to CRSsNP and controls in Adelaide and Beijing, but increased in CRSsNP compared to CRSwNP and controls in Tochigi. IFN-γ was increased in CRSsNP compared to CRSwNP and controls in Beijing but did not differ in other regions. TGF-β1 was increased in CRSsNP compared to CRSwNP in Benelux, Berlin, and Chengdu.
Wang et al^69^	2016 China	13 nonatopic CRSsNP, 9 atopic CRSsNP, 11 controls	Ethmoid mucosa, inferior turbinate	IL-4, IL-5, IL-6, IL-12, IL-13, IL-17A, IL-8, IFN-γ, eotaxin-1, MPO, RANTES, and CXCL10	IFN-γ increased in CRSsNP compared to controls. IL-5, IL-13, and eotaxin-1 increased in atopic CRSsNP compared to nonatopic CRSsNP and controls. No difference in IL-12, IL-4, IL-6, IL-17A, IL-8, MPO, RANTES, and CXCL10 between groups.
Kim et al ^56^	2016 Korea	70 CRSwNP, 63 CRSsNP, 20 controls	Nasal polyp, uncinate process	IL-5, IL-17A, IL-23. IFNy, CCL-11, CXCL-8	IL-17A and IL-23 negatively correlated with age in CRSwNP. No age-related changes for CXCL-1, CXCL-2, and CXCL-8 in CRSwNP.
Chen et al^48^	2017 China	42 CRSwNP 11 CRSsNP 13 controls	Nasal polyp, uncinate process	IL-4, IL-5, IL-9, IL-25, IL-33, IFN-γ, eotaxin-1, eotaxin-2, eotaxin-3, IL-8, IL-10, TSLP, MCP-4, TNF-a, ENA-78, RANTES, and TARC	IL-25 and eotaxin-1 were increased in nasal polyp tissue for CRSwNP compared to CRSsNP and controls. IL-4 and ENA-78 were increased in uncinate process tissue for CRSwNP compared to CRSsNP and controls. IL-5, IL-13, eotaxin-2, eotaxin-3, and IFN-g did not differ between CRSwNP, CRSsNP, and controls.
Ozturan et al ^32^	2017 Turkey	20 CRSwNP 20 CRSsNP 20 controls	Nasal polyp, uncinate process, inferior turbinate	IL-25 and IL-33	IL-25 showed no difference between CRS groups and controls. IL-33 was reduced in CRSwNP compared to CRSsNP and controls.
Min et al^45^	2017 USA	51 CRSwNP, 18 CRSsNP, 7 controls	Uncinate process, nasal polyp, inferior turbinate	IL-4, IL-13, Eotaxin-1, Eotaxin-2, Eotaxin-3	IL-13, Eotaxin-2, and Eotaxin-3 increased in CRSwNP compared to controls.
Dilidaer et al^70^	2017 China	25 CRSwNP, 12 CRSsNP, 10 controls	Nasal polyp, ethmoid mucosa, inferior turbinate	BAFF, IL-5	BAFF and IL-5 increased in CRSwNP compared to controls.
Jang et al^71^	2018 South Korea	19 ECRS 12 Non-ECRS 7 controls	Nasal polyp, uncinate process	IFN-a, CXCL-10/IP-10, IL-8, IL-4, IL-5, IL-13, IFN-B, and CCL11	IFN-B, IL-5, IL-13, and CCL11 were increased in ECRS compared to non-ECRS and controls. No differences between groups for IL-4 and CXCL10. IL-8 was increased in non-ECRS compared to ECRS and controls. IFN-a was undetectable in all groups.
Chen et al^72^	2018 China	132 CRSwNP 55 CRSsNP 50 controls	Nasal polyp, osteomeatal complex mucosa, inferior turbinate	IL-17A, MMP-7, MMP-9	IL-17A, MMP-7, and MMP-9 were increased in CRSwNP and CRSsNP compared to controls. IL-17A was increased in CRSwNP compared to CRSsNP.
Tang et al^73^	2018 China	33 ECRS 37 Non-ECRS 28 controls	Nasal polyp, inferior turbinate	IL-1β, IL-5, and IL-25	IL-1β, IL-5, and IL-25 were increased in ECRS and non-ECRS compared to controls, and increased in ECRS compared to non-ECRS.
Pulsipher et al ^58^	2018 USA	25 CRSwNP 28 CRSsNP 17 controls	Ethmoid mucosa	S100A12 and human neutrophil elastase	S100A12 and human neutrophil elastase were increased in CRSsNP compared to CRSwNP and controls. Levels of S100A12 were correlated to disease severity by CT scores but not QoL scores.
Chen et al^53^	2018 China	22 ECRSwNP 14 Non-ECRSwNP 23 CRSsNP 15 controls	Nasal polyp, uncinate process, middle turbinate	BAFF, IFN-γ, IL-4, IL-5, IL-13, and IL-17A	BAFF, IFN-γ, IL-4, IL-5, IL-13, and IL-17A were increased in CRSwNP compared to CRSsNP and controls. IL-5 and IL-13 were increased in ECRSwNP compared to non-ECRSwNP.
Kim et al^59^	2018 South Korea	69 ECRS 71 Non-ECRS 20 controls	Nasal polyp, uncinate process	IL-5, IL-17A, and IFN-γ	IL-5 was increased in severe and moderate ECRS compared with non-ECRS and controls. IL-17A was decreased in severe ECRS compared with mild ECRS and non-ECRS. IFN-γ was decreased in severe ECRS compared to moderate and mild ECRS.
Lin et al^52^	2018 China	11 CRSwNP 11 CRSsNP 8 controls	Nasal polyp, inferior turbinate, ethmoid mucosa	ECP, MPO, IL-25, IL-33, IL-13, IFN-γ, and IL-17	ECP, MPO, IL-25, IL-33, IL-5, IL-13, IFN-γ, and IL-17 were increased in CRSwNP and CRSsNP groups compared to controls. MPO, IFN-γ, and IL-17 were increased in CRSwNP compared to CRSsNP. ECP, IL-25, IL-33, IL-5, and IL-13 did not differ between CRSwNP and CRSsNP.
Wei et al^74^	2018 China	63 CRSwNP 25 controls	Nasal polyp, uncinate process	Periostin and TSLP	Periostin and TSLP were increased in ECRSwNP compared to non-ECRSwNP.
Dogan et al^75^	2018 Turkey	33 CRSwNP, 29 controls	Sinonasal tissue	AREG, IL-19, IL-21, IL-25, IL-33, TSLP	AREG, IL-19, IL-21, IL-25, IL-33, TSLP increased in CRSwNP compared to controls.
Li et al ^76^	2019 China	51 ECRSwNP 48 Non-ECRSwNP 50 CRSsNP 58 controls	Nasal polyp, uncinate process, inferior turbinate, sinonasal mucosa	ECP, eotaxin, IL-4, IL-5, IL-13, and IFN-γ	ECP, eotaxin, IL-4, IL-5, and IL-13 were increased in ECRSwNP compared to non-ECRSwNP, CRSsNP, and controls. IFN-γ did not differ between groups.
Yan et al^77^	2019 China	192 ECRSwNP 52 Non-ECRSwNP 40 controls	Nasal polyp, inferior turbinate, uncinate process	IL-5, IL-17A, IFN-γ, GM-CSF, CXCL8, and TNF-a	IL-5, GM-CSF, and IFN-γ were increased in ECRSwNP compared to controls. CXCL8, IL-17A, and IFN-g were increased in non-ECRSwNP compared to controls.
Kim et al^54^	2019 South Korea	13 CRSwNP 57 CRSsNP 10 controls	Uncinate process	IL-1α, IL-1β, IL-2Rα, IL-4, IL-5, IL-6, IL-8, IL-10, IL-13, IL-17A, IL-22, IL-23, IFN-γ, TNF-α, CCL-11, CCL-13, CCL-24, RANTES, CXCL-1, CXCL-2, CXCL-8, MPO, VCAM-1, ICAM-1, MMP-1, MMP-2, MMP-3, MMP-7, MMP-9, TIMP-1, and TGF-β1	ECP and CCL-24 were increased in CRSwNP compared to CRSsNP. IL-17A, CXCL-2, and IFN-γ were increased in CRSsNP compared to CRSwNP. ECP, MPO, IL-4, IL-1, CCL-11, CCL-24, total IgE, IL-1α, IL-6, IL-8, MMP-1, MMP-7, MMP-9, and CXCL-1 were increased in CRSwNP compared to controls.IL-4, IL-13, CCL-11, CCL-24, ECP, total IgE, IL-1α, IL-6, IL-8, CXCL-1, CXCL-2, MPO, IL-17A, IL-22, total IgE, TGF-β1, MMP-1, MMP-7, MMP-9, and TNF-α were increased in CRSsNP compared to controls. CXCL-2, CXCL-8, and MMP-9/TIMP-1 were significantly correlated with disease extent in CRSsNP.
Zhang et al^78^	2019 Belgium	21 CRSwNP 8 controls	Nasal polyp, inferior turbinate	IL-4, IL-5, IL-13 IL-17, IFN-γ, MPO, MCP-1, MUC5AC, and MUC5B	IL-4, IL-5, IL-13, MUC5AC, and MUC5B were increased in CRSwNP compared to controls. IFN-γ and IL-17 did not differ between CRSwNP and controls.
Ryu et al^79^	2019 South Korea	70 primary CRSwNP 86 refractory CRSwNP 23 controls	Nasal polyp, uncinate process	BAFF, CCL-11, CCL-24, IL-5, IL-8, IL-13, IL-17A, IL-23, IFN-γ, MPO, MMP-1, MMP-2, MMP-3, MMP-7, MMP-9, TIMP-1. TGF-β1	IL-5, ECP, CCL-11, CCL-24, and IL-13 were increased in primary and refractory CRSwNP compared to controls. IFN-γ, BAFF, MPO, IL-8, IL-17A, and IL-23 were increased in refractory CRSwNP compared to primary CRSwNP. TGF-β1 was reduced in primary and refractory CRSwNP compared to controls, and reduced in refractory CRSwNP compared to primary CRSwNP. MMP2/TIMP1 and MMP9/TIMP1 ratios were increased in refractory CRSwNP compared to controls and primary CRSwNP.
Tian et al^80^	2019 China	12 ECRSwNP 10 non-ECRSwNP 9 controls	Nasal polyp, inferior turbinate	CCL26	CCL26 was increased in ECRSwNP compared with controls. There was no difference between non-ECRSwNP and controls.
Nakayama et al ^81^	2019 Japan	71 CRSwNP 13 controls	Nasal polyp, uncinate process	IL-5, IL-13, IL-17, eotaxin-3/CCL26, IFN-γ, periostin, eotaxin/CCL11, eotaxin-2/CCL24, G-CSF, IL-8, IL-10	Eotaxin-2/CCL24 was increased in CRSwNP compared to controls. IL-5, Eotaxin/CCL11, Eotaxin-3/CCL26, Periostin, G-CSF, IL-8, and IL-10 did not differ between CRSwNP and controls.
Kim et al^82^	2019 South Korea	21 ECRSwNP (nasal polyp) 15 non-ECRSwNP (nasal polyp) 16 ECRSwNP (uncinate process) 11 non-ECRSwNP (uncinate process) 20 CRSsNP 9 controls	Nasal polyp, uncinate process	IL-1α, IL-1β, IL-2Rα, IL-4, IL-5, IL-6, IL-10, IL-13, IL-17A, IL-22, IL-23, IFN-γ, TNF-α, CCL-11, CCL-24, RANTES, CXCL-1, CXCL-2, CXCL-8, MPO, MCP-4, VCAM-1, ICAM-1, MMP-1, MMP-2, MMP-3, MMP-9, TIMP-1	IL-4, IL-5, IL-13, CCL-11, CCL-24, MCP-4, RANTES, IL-17A, IL-23, CXCL-1, CXCL-2, CXCL-8, IFN-γ, IL-1α, IL-1β, IL-2Rα, IL-6, ICAM-1, MMP-1, and MMP-9 were increased in non-ECRSwNP nasal polyp compared to controls. TIMP-1 was decreased in non-ECRSwNP compared to controls. IL-4, IL-5, IL-13, CCL-11, CCL-24, MCP-4, IL-1α, IL-1β, IL-2Rα, IL-6, VCAM-1, ICAM-1, IL-23, MMP-1, MMP-3, and MMP-9 were increased in ECRSwNP nasal polyp compared to controls. IL-5, IL-13, IL-17A, IL-22, IL-23, CXCL-2, ICAM-1, and MMP-9 were increased in CRSsNP compared to controls.
Kim et al^83^	2020 South Korea	157 CRSwNP 65 CRSsNP 22 controls	Nasal polyp, uncinate process	IL-22	IL-22 was increased in CRSsNP compared to CRSwNP and controls. IL-22 showed no difference between CRSwNP and controls.
Wang et al^84^	2020 China	65 CRSwNP with asthma 99 CRSwNP without asthma 31 controls	Nasal polyp	Total IgE	IgE was increased in CRSwNP with asthma group compared to CRSwNP without asthma and controls.
Ryu et al^55^	2020 South Korea	81 ECRSwNP, 113 non-ECRSwNP 86 CRSsNP 29 Controls	Nasal polyp, uncinate process	CCL-11, CCL-24, IL-5, periostin, CXCL-1, CXCL-2, CXCL-8, IL-17A, IL-22, IL-23, IL-6, IFN-γ, S100A8, IL-1ɑ, IL-1β, IL-10, IL-33, IL-25, BAFF, TGF-β1	IL-5, CCL-11, CCL-24, IFN-γ, and periostin increased with ageing in ECRSwNP, non-ECRSwNP, and CRSsNP compared to controls. IL-17A, CXCL-8, and IL-6 decreased with ageing in ECRSwNP, non-ECRSwNP, and CRSsNP compared to controls. CXCL-1 decreased with ageing in non-ECRSwNP and CRSsNP compared to controls.
Yao et al^85^	2020 China	12 ECRSwNP, 18 non-ECRSwNP 10 controls	Nasal polyp, inferior turbinate mucosa	Eotaxin-2, eotaxin-3, CCL18, IL-4, IL-5, IL-10, IL-12p70, IL-13, IL-15, CCL20, resistin, TGF-β2, TREM-1, CD14, GITR, and lipocalin-2	Eotaxin-2, eotaxin-3, CCL18, IL-4, IL-5, IL-10, IL-12p70, IL-13 and IL-15 were increased in ECRSwNP compared to non-ECRSwNP and controls. CCL20, resistin, TGF-β2, TREM-1, CD14, GITR, and lipocalin-2 were increased in non-ECRSwNP compared to ECRSwNP and controls.
Luo et al^86^	2020 China	60 CRSwNP 20 controls	Nasal polyp, inferior turbinate	IL-33, IL-4, IL-5, IL-17	IL-33 was increased in ECRSwNP and non-ECRSwNP compared with controls. IL-4 and IL-5 were increased in ECRSwNP compared to non-ECRSwNP. IL-17 was increased in non-ECRSwNP compared with ECRSwNP.
Shin et al^87^	2020 South Korea	14 ECRSwNP 16 non-ECRSwNP 8 controls	Nasal polyp, uncinate process	IL-6, IL-10, IL-25, IL-33, TSLP	IL-25, IL-33, and TSLP were increased in ECRSwNP compared with non-ECRSwNP and controls. TSLP was increased in non-ECRSsNP compared to controls.
Chang et al^88^	2020 China	134 CRSwNP, 67 CRSsNP, 62 controls	Nasal polyp, osteomeatal complex tissue	Eotaxin, ECP	Eotaxin and ECP increased in CRSwNP compared to CRSsNP and controls.
Lu et al^89^	2021 China	22 ECRSwNP 26 non-ECRSwNP 10 controls	Nasal polyp, uncinate process	SAA	SAA was increased in ECRSwNP and non-ECRSwNP compared with controls.
Wang et al^90^	2021 China	16 ECRSwNP 16 non-ECRSwNP 16 controls	Nasal polyp, middle turbinate mucosa	IL-1β	IL-1β was increased in ECRSwNP and non-ECRSwNP compared with controls. IL-1β was increased in ECRSwNP compared with non-ECRSwNP.
Lin et al^57^	2021 Taiwan	61 CRSwNP 26 controls	Nasal polyp, inferior turbinate mucosa, middle turbinate mucosa	p-JAK2, IL-2, IL-5, IL-6, IL-12, IL-13, G-CSF, GM-CSF, IFN-γ	p-JAK2, IL-5, IL-6, IL-13, IFN-γ, G-CSF, and GM-CSF were increased in CRSwNP compared with controls. p-JAK2, IL-5, IL-6, and G-CSF were correlated with LMK-CT and SNOT-22 scores.
Lucas et al^50^	2021 Brazil	34 CRSwNP 26 CRSsNP 26 controls	Nasal polyp, middle meatus mucosa	IL-6, IL-10, BMP-7, MMP-9, TGF-β	TGF-β was decreased in CRSwNP and increased in CRSsNP compared with controls.BMP-7 and MMP-9 showed no difference between CRSwNP, CRSsNP, and controls. IL-6 was increased in CRSwNP compared with CRSsNP and controls. IL-10 was decreased in CRSwNP and CRSsNP compared with controls.
Smith et al^47^	2021 USA	87 CRSwNP 64 CRSsNP 74 controls	Olfactory cleft mucus	CCL2, CCL3, CCL5, CCL11, CCL20, CXCL1, CXCL5, CXCL9, CXCL11, EGF, bFGF, IgE, IL-2, IL-4, IL-5, IL-6, IL-8, IL-9, IL-10, IL-13, IL-17A, IL-17F, IL-23, IL-33, TNFα, VEGF-A	CCL2, CCL3, CCL5, CCL11, CCL20, CXCL1, CXCL5, CXCL9, CXCL11, bFGF, IgE, IL-2, IL-4, IL-5, IL-6, IL-9, IL-10, IL-13, IL-17A, IL-17F, IL-23, IL33, and TNFα were increased in CRS compared with controls. IL-8 was reduced in CRS compared with controls. EGF and VEGF-A showed no difference between CRS and controls. CCL2, CCL3, CXCL11, IgE, IL-5, IL-9, and IL-13 were increased in CRSwNP compared with CRSsNP. IL-8 and VEGF-A were decreased in CRSwNP compared with CRSsNP.
Klingler et al ^40^	2021 USA	55 CRSwNP 126 CRSsNP 42 controls	Ethmoid mucosa	CXCL9, CXCL10, CSF3, SAA, IL-1β, IL-6	CXCL9 and CXCL10 were increased in T1 CRSsNP compared with non-T1 CRSsNP and controls. CSF3 was increased in T3 CRSsNP compared with controls. SAA, IL-1β, and IL-6 showed no difference between T3 CRSsNP and controls.
Vaitkus et al ^91^	2021 Lithuania	59 CRSwNP 52 controls	Nasal polyp, middle turbinate mucosa	IL-1β, IL-2, IL-4, IL-5, IL-6, IL-7, IL-10, IL-13, IL-21, and IL-22	Patients aged 18–30 years: IL-1, IL-2, IL-5, and IL-22 were increased in CRSwNP compared with controls, while IL-4, IL-6, IL-7, IL-10, IL-12, and IL-13 showed no difference. Patients aged 31–50 years: IL-2, IL-4, IL-5, and IL-22 were increased in CRSwNP compared with controls. Patients aged 51+ years: IL-2, IL-4, and IL-22 were increased in CRSwNP compared with controls.

Abbreviations: 1,25-OH-D3, calcitriol; 25-OH-D3, calcifediol; AERD, aspirin-exacerbated respiratory disease; AREG, amphiregulin; *A. flavus*, *Aspergillus flavus*; AFRS, allergic fungal rhinosinusitis; BAFF, B-cell activating factor; BCA, B cell-attracting chemokine; bFGF, basic fibroblast growth factor; BMP, bone morphogenetic protein; CCL, C-C motif chemokine ligand; CD, cluster of differentiation; CRS, chronic rhinosinusitis; CRSsNP, chronic rhinosinusitis without nasal polyposis; CRSwNP, chronic rhinosinusitis with nasal polyposis; CSF, colony stimulating factor; CT, computed tomography; CXCL, C-X-C motif chemokine ligand; ECP, eosinophilic cationic protein; ECRS, eosinophilic chronic rhinosinusitis; ECRSwNP, eosinophilic chronic rhinosinusitis with nasal polyposis; EGF, epidermal growth factor; eMBP, eosinophil major basic protein; ENA, extractable nuclear antigen; G-CSF, granulocyte colony stimulating factor; GITR, glucocorticoid induced tumor necrosis factor related protein; GM-CSF, granulocyte macrophage colony stimulating factor; ICAM, Intercellular adhesion molecule; IFN-γ, interferon gamma; IFN-β, interferon beta; Ig, immunoglobulin; IL, interleukin; IL-2Rα, interleukin 2 subunit receptor alpha; IP, Interferon gamma-induced protein; LMK-CT, Lund–Mackay computed tomography; LT, leukotriene; MCP, monocyte chemoattractant protein; MIF, macrophage migration inhibitory factor; MIP, macrophage inflammatory protein; MMP, matrix metalloproteinase; MPO, myeloperoxidase; MUC, mucin; NERD, non-steroidal anti-inflammatory drug-exacerbated respiratory disease; Non-ECRS, non-eosinophilic chronic rhinosinusitis; non-ECRSwNP, non-eosinophilic chronic rhinosinusitis with nasal polyposis; non-T1, non-type 1 inflammation; PGE2, prostaglandin E2; p-JAK, phosphorylated janus activating kinase; QoL, quality of life; RANTES, regulated upon activation, normal T cell expressed and presumably secreted (CCL5); S100A12, S100 calcium-binding protein A12; S100A7, calcium binding protein A7; S100A8, calcium binding protein A8; SAA, serum amyloid A; SAE-IgE, immunoglobulin E antibodies to *Staphylococcus aureus* enterotoxin; SCF, stem cell factor; SDF, stromal cell derived factor; SDC-1, syndecan 1; sgp130 , soluble glycoprotein 130; sIgA , secretory immunoglobulin A; SNOT-22, 22-item sinonasal outcome test; SOL-IL-5Ra, soluble interleukin 5 receptor alpha; SP, surfactant protein; SPLUNC1, short-palate lung and nasal epithelial clone 1; T1, type 1 inflammation; T3, type 3 inflammation; TARC, thymus and activation-regulated chemokine; TGF-β, transforming growth factor beta; TIMP, tissue inhibitor of metalloproteinases; TNF-a, tumor necrosis factor alpha; TREM, triggering receptor expressed on myeloid cells; TSLP, thymic stromal lymphopoietin; uLTE4, urinary leukotriene E4; VCAM, vascular cellular adhesion molecule; VEGF, vascular endothelial growth factor; YKL-40, chitinase-3-like protein 1.

Control tissue was most often harvested from the uncinate process, although one study used olfactory cleft mucosa.^47^ Biomarker expression was greatest in nasal polyp tissue in most cases when compared to other tissue sites, especially for IL-25 and eotaxin-1.^48^

While Liu et al^49^ and Stevens et al^44^ found IL-10 to be increased in patients with CRSwNP compared to controls, Lucas et al found IL-10 was higher in controls.^50^ Similarly, Kim et al^51^ and Lin et al^52^ found IL-33 to be increased in patients with CRSwNP compared to controls, while Ozturan et al found the opposite.^32^ When comparing CRSwNP patients with CRSsNP, IL-17A and interferon gamma (IFN-γ) were found to be increased by Chen et al,^53^ but the reverse was found by Kim et al.^54^

A study of 309 patients by Ryu et al found that IL-5, CCL-11, CCL-24, IFN-γ, and periostin increased with ageing in CRS phenotypes when compared with controls, while IL-17A, CXCL-8, and IL-6 decreased with ageing.^55^ Study quality was strengthened by a large sample size and adjustment of outcomes for confounding factors such as atopy status, smoking history, and disease duration. Similarly, Kim et al, in a study of 70 patients with CRSwNP, found IL-17A and IL-23 to be negatively correlated with age.^56^ Wang et al conducted a multi-centre study of 573 patients across 6 countries in Europe, Asia, and Australia and found geographical variation in biomarkers.^17^ IL-17 was increased in CRSwNP compared to CRSsNP and controls in Adelaide and Beijing, but increased in CRSsNP compared to CRSwNP and controls in Tochigi. IFN-γ was increased in CRSsNP compared to CRSwNP and controls in Beijing but did not differ in other regions. IL-8 was increased in CRSwNP compared to controls in all regions. Study quality was good due to the multi-center design, clear inclusion criteria, and standardized sampling procedures.

### Correlation to Disease Severity

Lin et al studied 61 patients with CRSwNP and found that phospho-janus kinase 2 (p-JAK2), IL-5, IL-6, and granulocyte-colony stimulating factor (G-CSF) were correlated with LMK-CT and SNOT-22 scores.^57^ Patients were categorized into “mild” and “severe” CRS based on Lund–Kennedy score alone, and data on patients with CRSsNP was limited. Similarly, Pulshiper et al studied 70 patients and found that levels of S100 calcium-binding protein A12 (S100A12) were increased in CRSsNP compared to CRSwNP and were correlated to LMK-CT scores but not to the rhinosinusitis disability index, a quality of life score.^58^ Clear CRS inclusion criteria were not specified and healthy controls included patients with nasal obstruction with associated quality of life implications. Kim et al stratified 69 patients into mild, moderate, and severe ECRSwNP using the Japanese Epidemiological Survey of Refractory Eosinophilic Chronic Rhinosinusitis score, which considers clinical examination findings, CT results and eosinophil counts.^59^ They found that IL-17A and IFN-γ were decreased in severe ECRSwNP compared with mild ECRSwNP. Inclusion criteria were clear and each subgroup had a minimum of 13 patients, but subgroups were not matched for sex or asthma status.

### Urine Biomarkers

One urine biomarker was identified in a USA study of 115 patients with unspecified CRS and 38 controls by Santarelli et al.^19^ Urinary leukotriene E4 (uLTE4) was increased in patients with CRS compared to controls. Elevated uLTE4 levels were correlated with the presence of comorbid asthma but not with atopy. Subgroup analyses for other CRS phenotypes were not performed.

## Discussion

This review summarizes the expression pattern among CRS phenotypes of 143 biomarkers identified from studies of nasal tissue, nasal secretions, nasal lavage fluid, serum, or urine. Biomarker profiles are presented to distinguish patients with CRSwNP, CRSsNP, ECRSwNP, non-ECRSwNP, refractory CRS, and primary CRS from each other or from controls. Biomarkers such as serum IgE, IL-17, and pentraxin-3 or nasal p-JAK2, IL-5, IL-6, IL-17A, G-CSF, and IFN-γ are identified as correlated with disease severity. Studies by Ryu et al,^55^ Kim et al,^82^ and Wang et al^17^ identify biomarker profiles to vary by patient age, site of nasal tissue sample, and geographical patient group, respectively.

Previous systematic reviews have concentrated on specific biomarkers alone, such as periostin^92^ or matrix metalloproteinases^93^ while this review integrates findings from all available biomarkers. Additionally, this review is not limited to one specific CRS phenotype but compares data across multiple phenotypes. Limitations include the lack of studies from developing economies, which could reduce the generalizability of results to these settings. Additionally, patients from South America and Africa were not well represented among the included studies. However, studies are presented from 22 countries across six continents, and the majority of studies were conducted in countries where English is not the national language. This review also only considered studies which used ELISA or Luminex for the analysis of samples. These methods are relatively easy to perform and cost-effective, but as a result studies using polymerase chain reaction assays, aptamer-based analyses or other techniques were not included. However, biomarkers which are not detectable across a range of modalities are less likely to translate into clinical practice. Finally, we did not choose to specify the use of a single CRS diagnostic criterion for inclusion of a study within our review. This is because the studies dated back to 2006, prior to the publication of more recent CRS diagnostic criteria, and we believe that using any one set of criteria may limit the geographical variability of included studies.

Studies from this review suggest that nasal tissue has the largest body of evidence for biomarker analysis. Most biomarkers found to distinguish CRS phenotypes such as ECRS and refractory CRS were found in nasal tissue, specifically nasal polyp. This has the advantage of being a consistent site of collection and can be harvested using local anaesthetic in some cases.

On the other hand, serum has the advantages of ease of collection and reproducible sampling. However, the range of serum biomarkers used to distinguish CRS phenotypes from each other was limited in this study. Serum biomarkers appear to have more of a role in differentiating CRS patients from controls, so further investigation into the role of serum biomarkers in disease identification could be considered.

Studies of nasal lavage fluid and nasal secretions were limited, and were hampered by inconsistencies in collection methods including site of sampling, amount of mucus sampled, and collection devices. This is significant as the proteome varies considerably throughout the nose. In particular, the role of immunoglobulins and eotaxin was limited in nasal secretions when compared to nasal tissue, and phenotypes such as ECRSwNP were not categorized within studies. Studies of urine and sputum were limited and warrant further investigation given the relative ease of collection of these samples, although biomarker profiles are likely to be less apparent when compared to nasal tissue.

Increasing evidence shows that earlier diagnosis and characterization of CRS is linked to better outcomes.^94^ Biomarkers are indicative of pathways which are important targets for biologic therapies currently under investigation for treating CRS, such as mepolizumab and omalizumab. Identifying a biomarker which can predict treatment response to these expensive therapies will be crucial to their uptake into clinical practice.^1^ Currently, specific protein biomarkers are not widely used in clinical practice and so further validation studies are required.

This review has identified multiple knowledge gaps in CRS biomarker research, such as the use of nasal lavage fluid and nasal secretions in distinguishing patients with ECRSwNP, the value of understudied nasal tissue collection sites such as olfactory cleft and maxillary sinus mucosa, and the prognostic role of nasal tissue biomarkers such as immunoglobulins. Specific biomarker targets for further research are also identified in those cytokines found to be associated with disease severity, such as p-JAK2, IL-5, IL-6, IL-17A, G-CSF, and IFN-γ. Additionally, future research should focus on exploring the conflicting results seen for nasal tissue biomarkers such as IFN-γ, IL-4, IL-10, IL-17A, and IL-33. This may have in part been due to geographical variation between studies or the small sample sizes of some included studies.

Future CRS biomarker studies can avoid the weaknesses of some of the studies in this review by considering measuring biomarkers at multiple time points and across multiple geographic regions, utilizing standardized outcome measures such as those proposed in EPOS2020,^1^ limiting the use of underpowered samples for biomarkers known to have reduced variation among sampling sites, specifying CRS inclusion criteria, and ensuring consistent reporting of the amount of sample harvested between patients.

Rather than relying on a single biomarker in isolation, CRS endotypes can be categorized by patient clusters with specific biomarker profiles.^95^ Future research should focus on exploring the interplay between biomarkers described in this review through prospective studies to identify correlations with treatment response. With monoclonal antibodies for CRS gaining increasing evidence of efficacy^96^ better means of identifying patients that should receive these expensive drugs is of paramount importance. Ultimately, a combination of several biomarkers will likely be the most promising approach in understanding the immunological mechanisms underlying the different phenotypes of CRS. Further studies of existing biomarkers should aim to bring patients closer to a personalized approach to CRS treatment.

## Supplemental Material

sj-docx-1-ajr-10.1177_19458924231190568 - Supplemental material for Systematic Review of Protein Biomarkers in Adult Patients With Chronic Rhinosinusitis Supplemental material, sj-docx-1-ajr-10.1177_19458924231190568 for Systematic Review of Protein Biomarkers in Adult Patients With Chronic Rhinosinusitis by Shyam A. Gokani, Andreas Espehana and Ana C. Pratas, Louis Luke, Ekta Sharma, Jennifer Mattock, Jelena Gavrilovic, Allan Clark, Tom Wileman, Carl M. Philpott in American Journal of Rhinology & Allergy

sj-docx-2-ajr-10.1177_19458924231190568 - Supplemental material for Systematic Review of Protein Biomarkers in Adult Patients With Chronic Rhinosinusitis Supplemental material, sj-docx-2-ajr-10.1177_19458924231190568 for Systematic Review of Protein Biomarkers in Adult Patients With Chronic Rhinosinusitis by Shyam A. Gokani, Andreas Espehana and Ana C. Pratas, Louis Luke, Ekta Sharma, Jennifer Mattock, Jelena Gavrilovic, Allan Clark, Tom Wileman, Carl M. Philpott in American Journal of Rhinology & Allergy
