# Supplementary material for: Systematic Review of Protein Biomarkers in Adult Patients With Chronic Rhinosinusitis
Source: Am J Rhinol Allergy. 2023 Jul 25;37(6):705–29. doi: 10.1177/19458924231190568 (PMC10548774; doi:10.1177/19458924231190568)
Supplement: sj-docx-1-ajr-10.1177_19458924231190568 - Supplemental material for Systematic Review of Protein Biomarkers in Adult Patients With Chronic Rhinosinusitis [file sj-docx-1-ajr-10.1177_19458924231190568.docx]

Supplementary file 1 – Search strategy

Search strategy for MEDLINE via Ovid SP

1. Sinus.mp. or sinusitis.tw,kw
2. rhinitis.tw,kw.
3. aspirin exacerbated respiratory disease.tw,kw.
4. AERD.tw,kw.
5. CRS*.tw,kw.
6. ECRS*.tw,kw.
7. rhinosinusitis.tw,kw.
8. non-steroidal anti-inflammatory drug exacerbated respiratory disease.tw,kw.
9. NSAID exacerbated respiratory disease.tw,kw.
10. NERD.tw,kw.
11. (nose adj2 polyp*).tw,kw.
12. (nasal adj2 polyp*).tw,kw.
13. exp Rhinitis/ or exp Sinusitis/ or exp Nasal Polyps/
14. (chronic adj2 rhinosinusitis).mp.
15. (chronic adj2 sinusitis).mp.
16. or/1-15
17. ELISA.tw,kw.
18. Luminex.tw,kw.
19. (enzyme linked adj2 assay).tw,kw.
20. multiplex.tw,kw.
21. exp immunoenzyme techniques/ or exp immunosorbent techniques/
22. (abbott or roche or siemens or danaher or diasorin molecular or sysmex or biomerieux or qiagen or thermofisher or agilent).tw,kw.
23. 17 or 18 or 19 or 20 or 21 or 22
24. predict*.tw,kw.
25. biomarker*.tw,kw.
26. diagnos*.tw,kw.
27. endotyp*.tw,kw.
28. prognos*.tw,kw.
29. exp Biomarkers/
30. or/24-29
31. 16 and 23 and 30

Search Strategy for Web of Science

(((TS=(Sinus OR sinusitis OR rhinitis OR aspirin exacerbated respiratory disease OR AERD OR CRS* OR ECRS* OR rhinosinusitis OR non-steroidal anti-inflammatory drug exacerbated respiratory disease OR NERD OR (nose NEAR/2 polyp*) OR (nasal NEAR/2 polyp*))) AND TS=(ELISA OR Luminex OR (enzyme linked NEAR/2 assay) OR multiplex OR (abbott or roche or siemens or danaher or diasorin molecular or sysmex or biomerieux or qiagen or thermofisher or agilent))) AND TS=(predict* OR biomarker* OR diagnos* OR endotyp* OR prognos*)))
