# Supplementary material for: Systematic Review of Protein Biomarkers in Adult Patients With Chronic Rhinosinusitis
Source: Am J Rhinol Allergy. 2023 Jul 25;37(6):705–29. doi: 10.1177/19458924231190568 (PMC10548774; doi:10.1177/19458924231190568)
Supplement: sj-docx-2-ajr-10.1177_19458924231190568 - Supplemental material for Systematic Review of Protein Biomarkers in Adult Patients With Chronic Rhinosinusitis [file sj-docx-2-ajr-10.1177_19458924231190568.docx]

| **Study Reference** | (Hirschberg *et al.*, 2003) | (Ali *et al.*, 2005) | (Perez-Novo *et al.*, 2006) | (Van Zele *et al.*, 2007) | (Lackner *et al.*, 2007) | (Liu *et al.*, 2009) | (Gevaert *et al.*, 2009) | (Shi *et al.*, 2009) | (Li *et al.*, 2010) | (Peters *et al.*, 2010) | (Schmid *et al.*, 2010) | (Sejima *et al.*, 2012) | (Keseroglu *et al.*, 2012) | (Hulse *et al.*, 2013) | (Derycke *et al.*, 2014) | (Li *et al.*, 2014) |
| --- | --- | --- | --- | --- | --- | --- | --- | --- | --- | --- | --- | --- | --- | --- | --- | --- |
| 1. Was the research question clearly stated? | 1 | 1 | 1 | 1 | 1 | 1 | 1 | 1 | 1 | 1 | 1 | 0 | 1 | 1 | 0 | 1 |
| 2. Was the study population clearly specified and defined? | 0 | 0 | 1 | 1 | 1 | 1 | 1 | 1 | 1 | 1 | 1 | 0 | 1 | 1 | 0 | 0 |
| 3. Was the participation rate of eligible persons at least 50%? | 0 | 0 | 0 | 0 | 1 | 0 | 0 | 0 | 0 | 0 | 0 | 0 | 0 | 0 | 0 | 0 |
| 4. Were the subjects selected from similar populations? | 0 | 0 | 1 | 0 | 1 | 1 | 1 | 1 | 0 | 1 | 1 | 1 | 1 | 1 | 1 | 1 |
| 5. Was a sample size justification provided? | 0 | 0 | 0 | 0 | 0 | 0 | 0 | 0 | 0 | 0 | 1 | 0 | 0 | 0 | 0 | 0 |
| 6. Were the exposures measured prior to outcomes? | 1 | 1 | 1 | 1 | 1 | 1 | 1 | 1 | 1 | 1 | 1 | 1 | 1 | 1 | 1 | 1 |
| 7. Was the timeframe sufficient? | 1 | 1 | 1 | 1 | 1 | 1 | 1 | 1 | 1 | 1 | 1 | 1 | 1 | 1 | 1 | 1 |
| 8. Did the study examine different levels of exposure? | 1 | 0 | 1 | 0 | 1 | 0 | 0 | 0 | 0 | 0 | 1 | 0 | 0 | 0 | 0 | 0 |
| 9. Were exposures clearly defined, valid and reliable? | 0 | 1 | 1 | 1 | 1 | 1 | 1 | 1 | 1 | 1 | 1 | 1 | 0 | 1 | 0 | 1 |
| 10. Was the exposure assessed more than once over time? | 0 | 0 | 0 | 0 | 0 | 0 | 0 | 0 | 0 | 0 | 1 | 0 | 0 | 0 | 0 | 0 |
| 11. Were outcomes clearly defined, valid and reliable? | 0 | 1 | 1 | 1 | 1 | 1 | 1 | 1 | 1 | 1 | 1 | 1 | 0 | 1 | 1 | 1 |
| 12. Were assessors blinded to the exposures of participants? | 0 | 0 | 0 | 0 | 0 | 0 | 1 | 1 | 0 | 0 | 0 | 0 | 0 | 1 | 0 | 1 |
| 13. Was loss to follow-up after baseline 20% or less? | 0 | 0 | 0 | 1 | 0 | 1 | 1 | 1 | 1 | 1 | 0 | 1 | 0 | 0 | 1 | 1 |
| 14. Were potential confounding variables adjusted for? | 0 | 0 | 0 | 0 | 0 | 0 | 0 | 0 | 0 | 0 | 0 | 0 | 0 | 0 | 0 | 0 |
| Overall Quality Rating (good, fair or poor). | fair | poor | good | fair | good | fair | good | good | fair | fair | good | poor | fair | good | poor | fair |

| **Study Reference** | (Xiao *et al.*, 2015) | (Chao *et al.*, 2015) | (Cui *et al.*, 2015) | (Shin *et al.*, 2015) | (Stevens *et al.*, 2015) | (Tsybikov *et al.*, 2015) | (Schlosser *et al.*, 2016) | (Uhliarova *et al.*, 2016) | (Kim *et al.*, 2017) | (König *et al.*, 2016) | (Qin *et al.*, 2016) | (X. Wang *et al.*, 2016) | (B. Wang *et al.*, 2016) | (Kim *et al.*, 2016) | (Chen *et al.*, 2017) | (Ozturan *et al.*, 2017) |
| --- | --- | --- | --- | --- | --- | --- | --- | --- | --- | --- | --- | --- | --- | --- | --- | --- |
| 1. Was the research question clearly stated? | 1 | 0 | 1 | 0 | 1 | 0 | 1 | 1 | 1 | 0 | 1 | 1 | 1 | 0 | 1 | 1 |
| 2. Was the study population clearly specified and defined? | 1 | 1 | 1 | 1 | 1 | 1 | 1 | 1 | 1 | 0 | 1 | 1 | 1 | 1 | 1 | 1 |
| 3. Was the participation rate of eligible persons at least 50%? | 0 | 0 | 0 | 0 | 0 | 0 | 0 | 0 | 0 | 0 | 0 | 0 | 0 | 0 | 0 | 0 |
| 4. Were the subjects selected from similar populations? | 1 | 1 | 1 | 0 | 1 | 1 | 1 | 1 | 1 | 0 | 0 | 1 | 1 | 1 | 1 | 0 |
| 5. Was a sample size justification provided? | 0 | 0 | 0 | 0 | 0 | 0 | 0 | 0 | 0 | 0 | 0 | 0 | 0 | 0 | 0 | 0 |
| 6. Were the exposures measured prior to outcomes? | 1 | 1 | 1 | 1 | 1 | 1 | 1 | 1 | 1 | 1 | 1 | 1 | 1 | 1 | 1 | 1 |
| 7. Was the timeframe sufficient? | 1 | 1 | 1 | 1 | 1 | 1 | 1 | 1 | 1 | 1 | 1 | 1 | 1 | 1 | 1 | 1 |
| 8. Did the study examine different levels of exposure? | 1 | 1 | 0 | 0 | 1 | 0 | 1 | 0 | 1 | 0 | 0 | 0 | 0 | 1 | 1 | 0 |
| 9. Were exposures clearly defined, valid and reliable? | 1 | 1 | 1 | 1 | 0 | 1 | 1 | 1 | 1 | 0 | 1 | 1 | 0 | 1 | 1 | 1 |
| 10. Was the exposure assessed more than once over time? | 0 | 0 | 0 | 0 | 0 | 0 | 0 | 0 | 0 | 0 | 0 | 0 | 0 | 0 | 0 | 0 |
| 11. Were outcomes clearly defined, valid and reliable? | 1 | 1 | 1 | 1 | 1 | 1 | 1 | 1 | 1 | 1 | 1 | 1 | 1 | 1 | 1 | 1 |
| 12. Were assessors blinded to the exposures of participants? | 0 | 0 | 0 | 0 | 0 | 0 | 0 | 1 | 0 | 0 | 0 | 0 | 0 | 0 | 0 | 0 |
| 13. Was loss to follow-up after baseline 20% or less? | 1 | 1 | 1 | 1 | 1 | 1 | 0 | 0 | 1 | 1 | 1 | 0 | 0 | 0 | 0 | 0 |
| 14. Were potential confounding variables adjusted for? | 1 | 0 | 0 | 0 | 0 | 0 | 0 | 0 | 0 | 0 | 0 | 1 | 0 | 1 | 1 | 0 |
| Overall Quality Rating (good, fair or poor). | good | fair | good | fair | fair | fair | fair | good | fair | poor | poor | good | poor | fair | good | fair |

| **Study Reference** | (Min *et al.*, 2017) | (Dilidaer *et al.*, 2017) | (Maxfield *et al.*, 2018) | (Jang *et al.*, 2018) | (X. Chen *et al.*, 2018) | (Tang *et al.*, 2018) | (Pulsipher *et al.*, 2018) | (Rai *et al.*, 2018) | (K. Chen *et al.*, 2018) | (Kim *et al.*, 2018) | (Lin *et al.*, 2018) | (Wei *et al.*, 2018) | (Dogan, Sahin and Yenisey, 2019) | (Li *et al.*, 2019) | (Santarelli, Lam and Han, 2019) | (Yan *et al.*, 2019) |
| --- | --- | --- | --- | --- | --- | --- | --- | --- | --- | --- | --- | --- | --- | --- | --- | --- |
| 1. Was the research question clearly stated? | 1 | 1 | 1 | 1 | 1 | 0 | 1 | 1 | 0 | 1 | 0 | 1 | 1 | 0 | 1 | 0 |
| 2. Was the study population clearly specified and defined? | 1 | 1 | 0 | 1 | 1 | 1 | 0 | 0 | 1 | 1 | 1 | 1 | 1 | 1 | 1 | 1 |
| 3. Was the participation rate of eligible persons at least 50%? | 0 | 0 | 0 | 0 | 0 | 0 | 0 | 0 | 0 | 0 | 0 | 0 | 0 | 0 | 0 | 0 |
| 4. Were the subjects selected from similar populations? | 1 | 1 | 1 | 1 | 0 | 1 | 1 | 1 | 0 | 1 | 1 | 1 | 1 | 1 | 1 | 1 |
| 5. Was a sample size justification provided? | 0 | 0 | 1 | 0 | 0 | 0 | 0 | 0 | 0 | 0 | 0 | 0 | 0 | 0 | 0 | 0 |
| 6. Were the exposures measured prior to outcomes? | 1 | 1 | 1 | 1 | 1 | 1 | 1 | 1 | 1 | 1 | 1 | 1 | 1 | 1 | 1 | 1 |
| 7. Was the timeframe sufficient? | 1 | 1 | 1 | 1 | 1 | 1 | 1 | 1 | 1 | 1 | 1 | 1 | 1 | 1 | 1 | 1 |
| 8. Did the study examine different levels of exposure? | 1 | 1 | 1 | 1 | 0 | 1 | 1 | 0 | 0 | 1 | 0 | 1 | 0 | 0 | 1 | 0 |
| 9. Were exposures clearly defined, valid and reliable? | 1 | 1 | 0 | 1 | 1 | 1 | 1 | 1 | 1 | 1 | 1 | 1 | 1 | 1 | 1 | 1 |
| 10. Was the exposure assessed more than once over time? | 1 | 0 | 0 | 0 | 0 | 0 | 0 | 0 | 0 | 0 | 0 | 0 | 1 | 0 | 0 | 0 |
| 11. Were outcomes clearly defined, valid and reliable? | 1 | 1 | 1 | 1 | 1 | 1 | 1 | 1 | 1 | 1 | 1 | 1 | 1 | 1 | 1 | 1 |
| 12. Were assessors blinded to the exposures of participants? | 0 | 1 | 0 | 0 | 0 | 0 | 0 | 0 | 0 | 0 | 0 | 0 | 0 | 0 | 0 | 0 |
| 13. Was loss to follow-up after baseline 20% or less? | 0 | 0 | 0 | 0 | 0 | 0 | 0 | 0 | 0 | 0 | 0 | 0 | 0 | 0 | 0 | 0 |
| 14. Were potential confounding variables adjusted for? | 0 | 0 | 0 | 1 | 0 | 0 | 0 | 0 | 0 | 0 | 0 | 0 | 0 | 0 | 1 | 0 |
| Overall Quality Rating (good, fair or poor). | good | good | fair | good | fair | good | good | fair | poor | good | fair | poor | good | fair | good | fair |

| **Study Reference** | (D. W. Kim *et al.*, 2019) | (Zhang *et al.*, 2019) | (Ryu *et al.*, 2019) | (Tian *et al.*, 2019) | (Nakayama *et al.*, 2019) | (D. K. Kim *et al.*, 2019) | (D.-K. Kim *et al.*, 2019) | (Kim *et al.*, 2020) | (Wang *et al.*, 2020) | (Ryu *et al.*, 2020) | (Steiner *et al.*, 2020) | (Yao *et al.*, 2020) | (Luo *et al.*, 2020) | (Shin *et al.*, 2020) | (Candar *et al.*, 2020) | (Dutu *et al.*, 2018) |
| --- | --- | --- | --- | --- | --- | --- | --- | --- | --- | --- | --- | --- | --- | --- | --- | --- |
| 1. Was the research question clearly stated? | 1 | 0 | 1 | 1 | 1 | 1 | 1 | 1 | 1 | 1 | 1 | 1 | 1 | 1 | 1 | 1 |
| 2. Was the study population clearly specified and defined? | 1 | 1 | 1 | 1 | 1 | 1 | 1 | 1 | 1 | 1 | 1 | 1 | 0 | 1 | 1 | 1 |
| 3. Was the participation rate of eligible persons at least 50%? | 0 | 0 | 0 | 0 | 0 | 0 | 0 | 0 | 0 | 0 | 0 | 0 | 0 | 0 | 0 | 0 |
| 4. Were the subjects selected from similar populations? | 1 | 1 | 1 | 1 | 1 | 1 | 1 | 1 | 1 | 1 | 1 | 1 | 1 | 1 | 1 | 1 |
| 5. Was a sample size justification provided? | 0 | 0 | 0 | 0 | 0 | 0 | 0 | 0 | 0 | 0 | 0 | 0 | 0 | 0 | 0 | 0 |
| 6. Were the exposures measured prior to outcomes? | 1 | 1 | 1 | 1 | 1 | 1 | 1 | 1 | 1 | 1 | 1 | 1 | 1 | 1 | 1 | 1 |
| 7. Was the timeframe sufficient? | 1 | 1 | 0 | 0 | 0 | 0 | 1 | 0 | 1 | 0 | 0 | 0 | 0 | 0 | 0 | 0 |
| 8. Did the study examine different levels of exposure? | 1 | 0 | 1 | 1 | 1 | 1 | 1 | 1 | 0 | 1 | 1 | 1 | 0 | 1 | 1 | 0 |
| 9. Were exposures clearly defined, valid and reliable? | 1 | 1 | 1 | 1 | 1 | 1 | 1 | 1 | 1 | 1 | 0 | 1 | 0 | 1 | 1 | 1 |
| 10. Was the exposure assessed more than once over time? | 0 | 0 | 0 | 0 | 0 | 0 | 0 | 0 | 0 | 0 | 0 | 0 | 0 | 0 | 0 | 0 |
| 11. Were outcomes clearly defined, valid and reliable? | 1 | 1 | 1 | 1 | 1 | 1 | 1 | 1 | 1 | 1 | 1 | 1 | 1 | 1 | 1 | 1 |
| 12. Were assessors blinded to the exposures of participants? | 0 | 0 | 0 | 0 | 0 | 0 | 0 | 0 | 1 | 0 | 0 | 1 | 0 | 0 | 0 | 0 |
| 13. Was loss to follow-up after baseline 20% or less? | 0 | 0 | 0 | 0 | 0 | 0 | 0 | 0 | 0 | 0 | 0 | 0 | 0 | 0 | 0 | 0 |
| 14. Were potential confounding variables adjusted for? | 0 | 0 | 0 | 0 | 0 | 1 | 0 | 0 | 0 | 1 | 0 | 0 | 0 | 0 | 0 | 0 |
| Overall Quality Rating (good, fair or poor). | good | fair | fair | fair | poor | good | good | fair | fair | fair | fair | fair | poor | fair | fair | fair |

| **Study Reference** | (Gulluev *et al.*, 2021) | (Chang *et al.*, 2020) | (Lu *et al.*, 2021) | (Yuan *et al.*, 2021) | (Wen *et al.*, 2021) | (Wang *et al.*, 2021) | (Lin *et al.*, 2021) | (Lucas *et al.*, 2021) | (Hussien, Habieb and Hamdan, 2021) | (Smith *et al.*, 2021) | (Klingler *et al.*, 2021) | (Vaitkus *et al.*, 2021) | (Wang *et al.*, 2022) | (Shrestha *et al.*, 2022) |  |  |
| --- | --- | --- | --- | --- | --- | --- | --- | --- | --- | --- | --- | --- | --- | --- | --- | --- |
| 1. Was the research question clearly stated? | 1 | 1 | 1 | 1 | 1 | 1 | 1 | 1 | 1 | 1 | 1 | 1 | 1 | 1 |  |  |
| 2. Was the study population clearly specified and defined? | 0 | 1 | 1 | 1 | 1 | 1 | 1 | 1 | 1 | 1 | 1 | 1 | 1 | 1 |  |  |
| 3. Was the participation rate of eligible persons at least 50%? | 0 | 0 | 0 | 0 | 0 | 0 | 0 | 0 | 0 | 0 | 0 | 0 | 0 | 0 |  |  |
| 4. Were the subjects selected from similar populations? | 1 | 1 | 1 | 1 | 1 | 1 | 1 | 1 | 1 | 0 | 1 | 1 | 1 | 1 |  |  |
| 5. Was a sample size justification provided? | 0 | 0 | 0 | 0 | 0 | 0 | 0 | 0 | 0 | 0 | 0 | 0 | 0 | 0 |  |  |
| 6. Were the exposures measured prior to outcomes? | 1 | 1 | 1 | 1 | 1 | 1 | 1 | 1 | 1 | 1 | 1 | 1 | 1 | 1 |  |  |
| 7. Was the timeframe sufficient? | 1 | 1 | 0 | 0 | 0 | 0 | 0 | 0 | 0 | 0 | 0 | 0 | 1 | 0 |  |  |
| 8. Did the study examine different levels of exposure? | 0 | 0 | 1 | 1 | 1 | 1 | 1 | 0 | 0 | 0 | 1 | 1 | 1 | 1 |  |  |
| 9. Were exposures clearly defined, valid and reliable? | 1 | 1 | 1 | 1 | 1 | 1 | 1 | 1 | 1 | 1 | 1 | 1 | 1 | 1 |  |  |
| 10. Was the exposure assessed more than once over time? | 0 | 1 | 0 | 0 | 0 | 0 | 0 | 0 | 0 | 0 | 0 | 0 | 0 | 0 |  |  |
| 11. Were outcomes clearly defined, valid and reliable? | 1 | 1 | 1 | 1 | 1 | 1 | 1 | 1 | 1 | 1 | 1 | 1 | 1 | 1 |  |  |
| 12. Were assessors blinded to the exposures of participants? | 0 | 0 | 0 | 0 | 0 | 0 | 0 | 0 | 0 | 0 | 0 | 0 | 0 | 0 |  |  |
| 13. Was loss to follow-up after baseline 20% or less? | 0 | 0 | 0 | 0 | 0 | 0 | 0 | 0 | 0 | 0 | 0 | 0 | 1 | 0 |  |  |
| 14. Were potential confounding variables adjusted for? | 0 | 0 | 0 | 1 | 1 | 0 | 1 | 0 | 0 | 1 | 0 | 0 | 1 | 0 |  |  |
| Overall Quality Rating (good, fair or poor). | fair | fair | fair | fair | good | fair | good | fair | fair | fair | fair | fair | good | fair |  |  |

Key: 1 = yes. 0= no or cannot determine. N/A = not applicable.
